# Incidence and prevalence of immune-mediated extraintestinal manifestations in pediatric inflammatory bowel disease: a systematic review and meta-analysis

**DOI:** 10.1093/crocol/otag024

**Published:** 2026-03-31

**Authors:** Ruben J Colman, Sudheer K Vuyyuru, Virginia Solitano, John K MacDonald, Jessica Le, Amanda Ricciuto, Roberta Berard, Javed A Mohammed, Christopher Ma, Vipul Jairath, Eileen Crowley

**Affiliations:** Department of Pediatrics, Division of Pediatric Gastroenterology, Hepatology and Nutrition, Stanford University School of Medicine, Stanford, CA, United States; Department of Medicine, Division of Gastroenterology, Schulich School of Medicine, Western University, London, ON, Canada; Alimentiv Inc., London, ON, Canada; Division of Gastroenterology and Gastrointestinal Endoscopy, IRCCS Ospedale San Raffaele, Università Vita-Salute San Raffaele, Milan, Italy; Department of Epidemiology and Biostatistics, Western University, London, ON, Canada; Alimentiv Inc., London, ON, Canada; Alimentiv Inc., London, ON, Canada; Division of Gastroenterology, Hepatology and Nutrition, SickKids Inflammatory Bowel Disease Centre, The Hospital for Sick Children, Toronto, ON, Canada; Department of Paediatrics, University of Toronto, Toronto, ON, Canada; Department of Pediatrics, Division of Rheumatology, London Health Sciences Center, London, ON, Canada; Children’s Health Research Institute, London Health Sciences Centre, London, ON, Canada; Pediatric Dermatology, Department of Pediatrics, Children’s Hospital, London Health Sciences Center, London, ON, Canada; Alimentiv Inc., London, ON, Canada; Department of Community Health Sciences, Cumming School of Medicine, University of Calgary, Calgary, AB, Canada; Department of Medicine, Division of Gastroenterology and Hepatology, University of Calgary, Calgary, AB, Canada; Department of Medicine, Division of Gastroenterology, Schulich School of Medicine, Western University, London, ON, Canada; Alimentiv Inc., London, ON, Canada; Department of Epidemiology and Biostatistics, Western University, London, ON, Canada; Alimentiv Inc., London, ON, Canada; Department of Paediatrics, Division of Paediatric Gastroenterology, Hepatology and Nutrition, Children’s Hospital Western Ontario, Western University, London Health Sciences Centre, London, ON, Canada

**Keywords:** immune-mediated extraintestinal manifestations, pediatric inflammatory bowel disease, incidence, prevalence

## Abstract

**Background:**

Immune-mediated extraintestinal manifestations (IM-EIMs) are common in pediatric inflammatory bowel disease (pIBD). This systematic review and meta-analysis aimed to summarize the incidence and prevalence of IM-EIMs in pIBD.

**Methods:**

MEDLINE, EMBASE, CENTRAL, and clinicaltrials.gov were searched up to May 6, 2024, for studies that included pIBD (2-17 years) patients diagnosed with IM-EIMs. Primary outcomes included the incidence and prevalence of IM-EIMs among pIBD. Meta-analysis included pooled proportions of IM-EIMs using DerSimonian-Laird random-effects model.

**Results:**

The pooled proportion of IM-EIMs overall was 10% (95% CI, 8%-12%; I^2^ = 97.2%) among 65 pIBD studies (35 343 patients). The pooled proportion of IM-EIMs was 10% (95% CI, 7%-13%; I^2^ = 94.8%) among 37 Crohn’s disease (CD) studies (7657 patients), compared to 11% (95% CI, 7%-15%; I^2^ = 93.3%) among 39 ulcerative colitis (UC) studies (4698 patients). Enthesitis was the highest reported individual IM-EIM (23%, 95% CI, 4%-50%; I^2^ = 94.7%) in pIBD. Arthritis was the second highest reported individual IM-EIM (8%, 95% CI, 6%-10%; I^2^ = 96%) in pIBD, followed by primary sclerosing cholangitis (PSC) in UC (5%, 95% CI, 4%-7%; I^2^ = 81%). Uveitis, pyoderma gangrenosum, autoimmune hepatitis, and PSC in CD were the lowest reported IM-EIMs at 1% or less. The largest difference in proportions between IBD phenotypes was PSC, which was 4% higher in UC than CD.

**Conclusions:**

While musculoskeletal manifestations are common, fewer than 5% of pIBD patients experience ophthalmological, dermatological, and liver IM-EIMs. The small number of studies resulted in significant methodological and statistical heterogeneity. Multicenter collaborative efforts are needed to systematically describe the epidemiology of IM-EIMs in pIBD.

## Graphical Abstract

**Graphical Abstract:**
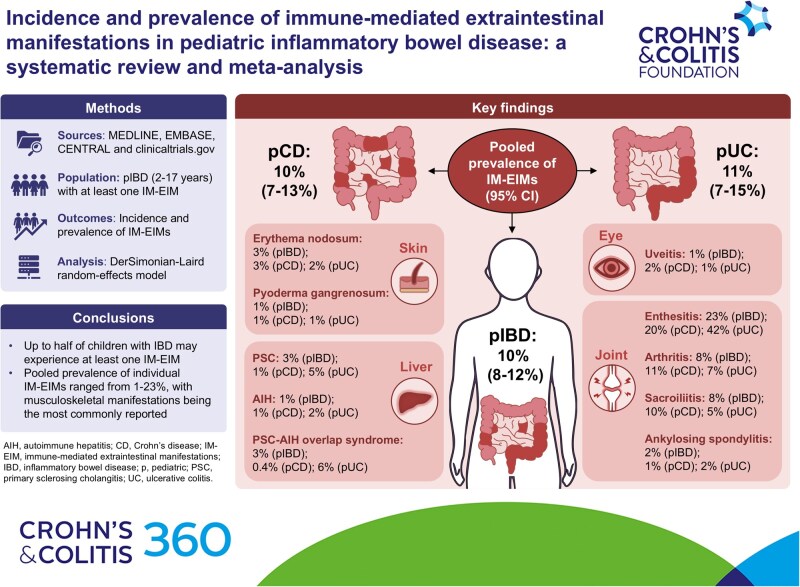

## Introduction

It is estimated that up to 50% of adult patients with inflammatory bowel disease (IBD) experience at least one extra-intestinal manifestation (EIM), which can present prior to, concurrently with, or after the diagnosis of IBD.^1^^,^^2^ These EIMs can involve nearly any organ system, including the musculoskeletal, dermatologic, hepatopancreatobiliary, ocular, renal, hematological, and pulmonary systems.^3^ EIMs can cause a significant challenge to patients and physicians and generally require multidisciplinary team care.

The epidemiology of EIMs in pediatric IBD is poorly characterized, despite higher reported rates of EIMs at IBD onset in pediatric patients compared to adult patients.^4–6^ At the time of IBD diagnosis, up to 28% of pediatric patients may present with at least one EIM, and up to 29% of pediatric patients will develop at least one EIM within the following 15 years.^4^^,^^6^ The presence or development of EIMs in pediatric IBD may be associated with a more severe disease course, with an increased risk of requiring biological treatment, surgical intervention, and escalation of medical therapies.^7^ EIMs can adversely affect a patient’s quality of life, and some EIMs, such as primary sclerosing cholangitis (PSC) and venous thromboembolism, can be life-threatening.

There is significant heterogeneity in the description of the pediatric IBD patient phenotypes who develop EIMs, as well as in the definitions used to describe EIMs.^8^ Nonspecific EIMs, such as hepatitis, anemia, pancreatitis, osteoporosis, glaucoma, nephrolithiasis, and arthralgia, have previously been excluded from definitions of EIMs, as these may represent adverse events of IBD therapy or complications from uncontrolled bowel inflammation rather than true immune-mediated (IM)-EIMs.^4^^,^^8^^,^^9^ Similarly, while psoriasis may be seen in pediatric IBD, it is often induced by anti-TNF therapy (i.e., paradoxical psoriasis), limiting its classification as a true IM-EIM of IBD.

The lack of standardized definitions has provided challenges and significant limitations in developing reproducible and generalizable findings from the primary studies, including clinical trials and prior systematic reviews. In adults with IBD, there have been recent efforts to standardize EIM definitions, including 3 combined systematic reviews/consensus statements.^10–12^ Among these, the first European Crohn′s and Colitis Organisation (ECCO) consensus guideline on EIMs in IBD considered a broad inclusion of the EIM definition, including disease and treatment-related complications.^10^ Two additional consensus studies focused on a more limited selection of EIMs including dermatological, musculoskeletal, and ophthalmologic manifestations only, in an effort to standardize definitions for future prospective studies.^11^^,^^12^ A similar consensus effort or recent systematic review of IM-EIMs in pediatric IBD has not yet been performed. The primary objective of this systematic review was to report the incidence and prevalence of IM-EIMs in pediatric IBD including musculoskeletal manifestations (i.e., axial and peripheral arthritis, sacroiliitis, ankylosing spondylitis, and enthesitis), ophthalmological manifestations (i.e., episcleritis, uveitis, and scleritis), dermatological manifestations (i.e., erythema nodosum, pyoderma gangrenosum, sweet syndrome, and granulomatous cutaneous lesions) and hepatobiliary manifestations (i.e., PSC, autoimmune hepatitis [AIH], granulomatous hepatitis, and PSC-AIH overlap syndrome; Table 1).

**Table 1:** Immune-mediated extraintestinal manifestations of IBD.

Organ system	Manifestations	Definitions
**Musculoskeletal**	Arthritis—axial/peripheral	Inflammatory reaction of axial (chest, back, hip) or peripheral (arms and legs) joints
	Sacroiliitis	Inflammation of sacroiliac joints
	Ankylosing spondylitis	Chronic axial inflammation with structural damage
	Enthesitis	Inflammation of the tendon-bone insertion site
**Ophthalmological**	Episcleritis	Self-limiting inflammation of the episclera with no threat to vision
	Uveitis	Sight-threatening intraocular inflammation of the uvea, including iris, ciliary body and choroid
	Scleritis	Severe, sight-threatening inflammation of the sclera with associated ocular complications
**Dermatological**	Erythema nodosum	Panniculitis presenting as painful erythematous subcutaneous nodules
	Pyoderma gangrenosum	Neutrophilic dermatosis presenting as rapidly progressing painful ulcers
	Sweet syndrome (acute febrile neutrophilic dermatosis)	Neutrophilic infiltrates presenting as tender papules, plaques, and nodules; often accompanied by systemic symptoms such as fever and neutrophilia
	Granulomatous cutaneous lesions	Noncaseating granulomatous inflammation of the skin
**Hepatobiliary**	PSC	Chronic progressive inflammation of the intrahepatic and/or extrahepatic bile ducts
	AIH	Chronic progressive inflammation of the liver with histologic evidence of interface hepatitis in combination with laboratory abnormalities in the absence of other causes of liver disease
	Granulomatous hepatitis	Rare manifestation with granulomas in the liver
	PSC-AIH overlap syndrome	Coexistence of both PSC and AIH features, such as interface hepatitis and bile duct abnormalities, along with overlapping imaging and laboratory findings

Abbreviations: AIH, autoimmune hepatitis; IBD, inflammatory bowel disease; PSC, primary sclerosing cholangitis.

## Methods

This systematic review is reported according to the Preferred Reporting Items for Systematic Reviews and Meta-Analyses (PRISMA) statement^13^ and was conducted following an *a priori* developed protocol (available upon request).

### Search strategy

MEDLINE, Embase, Cochrane Central Register of Controlled Trials (CENTRAL), and clinicaltrials.gov were searched from inception to May 6, 2024. The search strategies are reported in Supplementary Appendix S1. Date restrictions were not applied. The references of relevant articles retrieved from the electronic databases were hand-searched to ensure that all eligible studies were identified.

### Eligibility criteria

Randomized controlled trials (RCTs), prospective and retrospective cohort studies, case-controlled studies and cross-sectional studies involving pediatric patients (2-17 years old) with a confirmed diagnosis of IBD (i.e., Crohn’s disease [CD] or ulcerative colitis [UC]) and at least 1 IM-EIM as specified in Table 1 were eligible for inclusion. Studies were eligible if they reported incidence or prevalence for any of the IM-EIMs of interest. Conference abstracts were eligible for inclusion. Case reports, case series with fewer than 10 participants, review articles and RCT protocols were excluded. Studies that were not published in English were excluded.

### Screening and data extraction

Five authors (RJC, SKV, VS, JL and JKM) screened the search results independently and in duplicate. Data on study details (i.e., study design, treatment phase, first author, year of publication, journal, number of participants, study arms, intervention and comparator), participant characteristics (i.e., age, sex, disease type, disease duration, disease severity as measured by the Pediatric Crohn’s Disease Activity Index, Pediatric Ulcerative Colitis Activity Index, Physician Global Assessment, Mayo Clinic Score, concomitant diseases, and medications), outcomes, and risk of bias of the included studies were independently extracted in duplicate by 4 authors (RJC, VS, JL, JKM) using a standardized spreadsheet (Excel, Microsoft Corp., Redmond, WA). Discrepancies encountered during screening or data extraction were resolved by discussion and consensus, or recourse to a third author (EC or JKM). The Newcastle-Ottawa tool was used to assess the methodological quality of included cohort and cross-sectional studies.^14^

### Outcomes

The primary outcomes included the incidence and prevalence of each IM-EIM (as defined in Table 1)^15^ among pediatric IBD stratified by CD and UC if reported. The cumulative prevalence of any IM-EIM among pediatric IBD, CD, and UC patients was also calculated where appropriate.

### Data synthesis and analysis

Descriptive statistics were used to summarize the incidence and prevalence of EIMs in pediatric IBD.

We planned to use the DerSimonian-Laird random-effects model to pool prevalence and incidence rates. The Freeman-Tukey double arcsine square root transformation method was used to compute point estimates of prevalence and incidence rates and corresponding 95% confidence intervals (95% CIs).^16^^,^^17^ The random-effects model was chosen to provide an inference about the average prevalence rate in the population of studies from which the included studies were assumed to be a random selection. We acknowledge there are no specific tests to assess heterogeneity in proportional meta-analysis as the I^2^ statistic was developed in the context of comparative data,^18^ thus we report the I^2^ statistic and interpret it with caution. Tests to evaluate publication bias, such as Egger’s test, Begg’s test and funnel plots, were developed in the context of comparative data; the assumption of positive results being more often published is not necessarily true for proportional studies. Even though it is possible to conduct these tests for proportional meta-analysis, there is no evidence that proportional data sufficiently adjusts for these tests. It is not recommended to use these tests for proportional meta-analyses and advise that publication bias be assessed qualitatively.^18^ Therefore, we did not perform the tests for publication bias.

## Results

A total of 9326 records were identified from the literature search and other sources. After removing duplicates, 6167 records remained for screening. A total of 5851 records were deemed ineligible based on the information provided in the title and abstract, leaving 316 articles that were assessed for full-text review. Two hundred and twenty-five articles were excluded with reasons (Table S1), and 91 reports of 90 studies were included for qualitative and quantitative synthesis (Figure S1).

### Description of included studies

We included 12 prospective cohort studies, 65 retrospective cohort studies, 6 prospective cross-sectional studies, and 6 retrospective cross-sectional studies. One study utilized both prospective and retrospective cohort data. The characteristics of included studies and references are reported in Table S2.

### Risk of bias

The risk of bias assessment for included cohort and cross-sectional studies is reported in Table S3. Seventeen of 78 cohort studies had a low risk of bias, and 61 cohort studies had a high risk of bias. One of 12 cross-sectional studies had a low risk of bias, and 11 cross-sectional studies had a high risk of bias.

### Incidence and prevalence of immune-mediated EIMs in pediatric IBD, CD, and UC

#### Incidence of IM-EIMs in pediatric IBD

Two studies reported on the incidence of individual IM-EIMs. The pooling of incidence rates was not possible. Chandrakumar et al., 2019 reported that the incidence of PSC in pediatric IBD in Manitoba (Canada) was 3.95 events per 1000 person-years.^19^ Colletti et al., 2019 reported on the incidence of IM-EIMs in a multi-national pediatric IBD cohort (USA, Canada and Europe) exposed to tumor necrosis factor-alpha (TNF-α) antagonists compared with a population exposed to non- biologics.^20^ The incidence of PSC, AIH and arthritis was 0.2, 0.1, and 0.2 events per 1000 person-years, respectively, in the TNF-α exposed cohort compared to 0.2, 0.1, and 0.1 events per 1000 person-years, respectively, in the non-biologics cohort.

#### Prevalence of IM-EIMs in pediatric IBD

**Figure 1:**
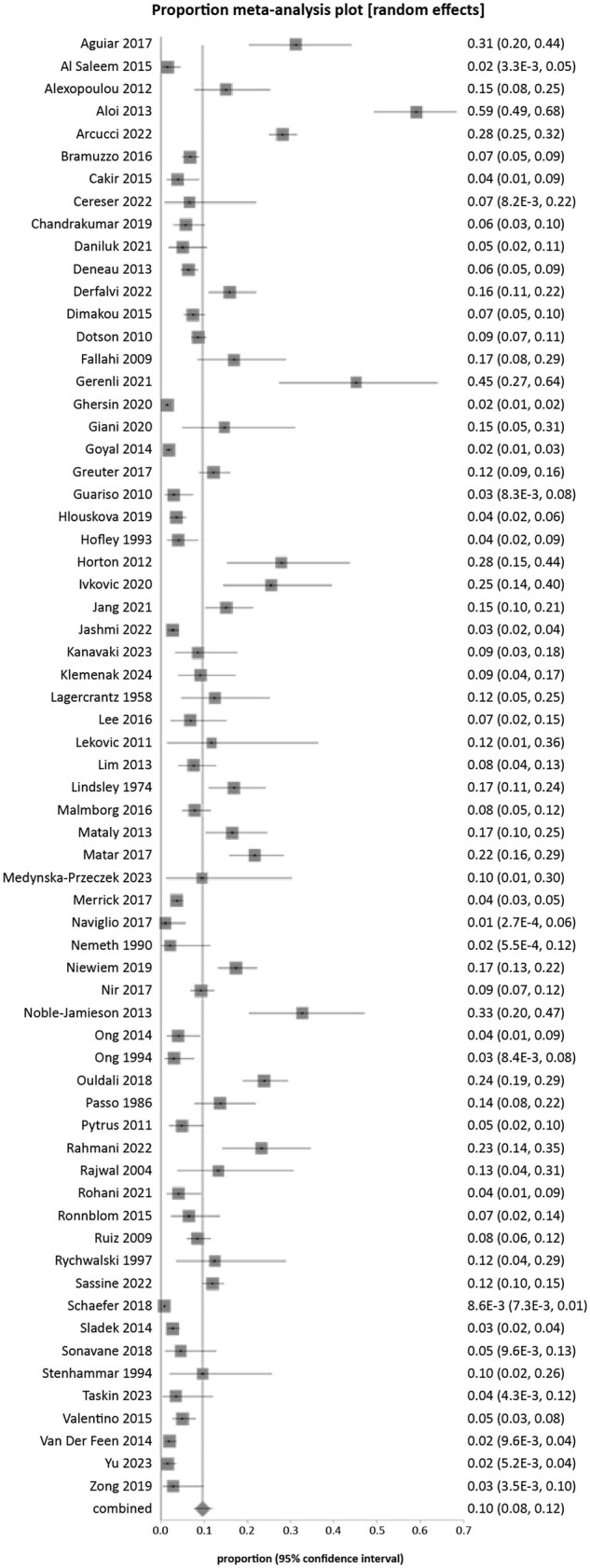
Pooled overall immune-mediated extra-intestinal manifestations in pediatric inflammatory bowel disease.

The cumulative prevalence of IM-EIMs in pediatric IBD ranged from a low of 0.9% (151/17 587) to a high of 45.2% (14/31). The overall pooled prevalence of IM-EIMs in pediatric IBD among 65 eligible studies including 35 343 patients was 10% (95% CI, 8%-12%, I^2^ = 97.2%; Figure 1). The prevalence of IM-EIMs in pediatric IBD as reported by the individual studies is presented in Table 2. The prevalence and incidence of individual (musculoskeletal, ophthalmological, dermatological, liver) IM-EIMs in pediatric IBD as reported by the individual studies is shown in Table 3.

**Table 2:** Prevalence of immune-mediated extra-intestinal manifestations in pediatric IBD, CD, and UC as reported by individual studies.

Study ID	Prevalence of IM-EIMs in IBD—% (*n*/*N*)	Prevalence of IM-EIMs in CD—% (*n*/*N*)	Prevalence of IM-EIMs in UC—% (*n*/*N*)
**1. Adamiak 2013^a^**	NA	NA	NA
**2. Afarideh 2024**	NR	NR	NR
**3. Aguiar 2017**	31.2 (20/64)	NR	NR
**4. Al Saleem 2015**	NR	NR	1.6 (3/188)
**5. Alexopoulou 2012**	15.1 (11/73)	NR	NR
**6. Aloi 2013**	NR	NR	59.1 (65/110)
**7. Alreheili 2018^b^**	NA	NA	NA
**8. Arcucci 2022**	28.2 (213/756)	31.2 (78/250)	26.7 (135/506)
**9. Ashton 2015^b^**	NA	NA	NA
**10. Ben Rabeh 2019^b^**	NA	NR	NR
**11. Bilgic Dagci 2022^b^**	NA	NA	NA
**12. Bramuzzo 2016**	6.8 (46/677)	NR	NR
**13. Cakir 2015**	3.9 (5/127)	7.0 (2/29)	3.3 (3/90)
**14. Cereser 2022**	NR	6.7 (2/30)	NR
**15. Chandrakumar 2019**	5.8 (11/190)	NR	10.0 (9/90)
**16. Cohen 2020^b^**	NA	NA	NA
**17. Colletti 2019**	NR	NR	NR
**18. Daniluk 2021**	5.0 (6/119)	NR	7.8 (6/77)
**19. Dass 2023**	NA	NR	NR
**20. Deneau 2013**	6.4 (39/607)	1.9 (6/317)	12.6 (33/262)
**21. Derfalvi 2022**	NR	35.4 (29/82)	NR
**22. Dimakou 2015**	7.4 (36/483)	12.6 (21/167)	4.1 (11/267)
**23. Dong 2023**	NR	NA	NR
**24. Dotson 2010**	8.6 (87/1009)	9.9 (72/728)	5.3 (15/281)
**25. Dzongowski 2023**	NA	NA	NA
**26. Fallahi 2009**	16.9 (10/59)	NR	NR
**27. Gerenli 2021**	45.2 (14/31)	54.5 (6/11)	41.2 (7/17)
**28. Gerenli 2022**	29.6 (8/27)	45.5 (5/11)	21.4 (3/14)
**29. Ghersin 2020**	1.5 (36/2372)	1.4 (22/1612)	1.8 (14/760)
**30. Giani 2020**	14.7 (5/34)	12.5 (4/32)	50.0 (1/2)
**31. Goyal 2014**	1.8 (29/1569)	0.8 (9/1075)	4.0 (20/494)
**32. Greuter 2017**	12.2 (40/329)	18.5 (32/173)	8.3 (13/156)
**33. Guariso 2010**	3.0 (4/133)	4.5 (3/67)	1.7 (1/58)
**34. Hlouskova 2019**	3.6 (15/412)	NR	NR
**35. Hofley 1993**	4.0 (6/147)	6.2 (6/97)	0 (0/50)
**36. Horton 2012**	27.9 (12/43)	NR	NR
**37. Isa 2018**	NR	NA	NR
**38. Ivkovic 2020**	25.5 (13/51)	21.1 (4/19)	18.8 (6/32)
**39. Jang 2021**	15.1 (26/172)	9.5 (13/137)	22.9 (8/35)
**40. Jang 2022**	NR	NR	NA
**41. Jashmi 2022**	2.7 (21/770)	NR	NR
**42. Jose 2009^b^**	NA	NR	NR
**43. Kanavaki 2023**	8.6 (6/70)	NA	NA
**44. Kim 2017**	NR	NR	NA
**45. Klemenak 2024**	9.2 (8/87)	NR	NR
**46. Kourti 2023**	NA	NA	NA
**47. Kwon 2022^b^**	NR	NR	NA
**48. Lagercrantz 1958**	NR	NR	12.5 (6/48)
**49. Lee 2016**	NR	6.8 (5/73)	NR
**50. Lekovic 2011**	NR	NR	11.8 (2/17)
**51. Levy 2019**	NA	NR	NR
**52. Lim 2013**	7.6 (12/157)	NR	NR
**53. Lindsley 1974**	16.9 (23/136)	5.8 (5/86)	36.0 (18/50)
**54. Malmborg 2016**	7.9 (22/280)	NR	NR
**55. Maniscalco 2024**	NR	NR	NR
**56. Mataly 2013**	NR	NR	16.5 (19/115)
**57. Matar 2017**	21.7 (38/175)	20.9 (27/129)	23.9 (11/46)
**58. Medynska-Przeczek 2023**	NR	9.5 (2/21)	NR
**59. Merrick 2017**	3.7 (30/809)	NR	NR
**60. Naviglio 2017**	1.1 (1/94)	2.2 (1/46)	0 (0/46)
**61. Nemeth 1990**	2.2 (1/46)	0 (0/12)	2.9 (1/34)
**62. Niewiem 2019**	17.4 (50/287)	15.7 (22/140)	19.0 (28/147)
**63. Nir 2017**	9.3 (40/430)	12.3 (37/301)	2.3 (3/129)
**64. Noble-Jamieson 2013**	NR	NR	32.7 (17/52)
**65. Ong 2014**	4.1 (5/123)	4.9 (4/82)	3.6 (1/28)
**66. Ong 1994**	3.1 (4/130)	2.1 (1/47)	3.6 (3/83)
**67. Ouldali 2018**	NR	23.9 (65/272)	NR
**68. Passo 1986**	13.7 (14/102)	15.5 (9/58)	9.1 (4/44)
**69. Pytrus 2011**	4.9 (7/143)	3.5 (3/86)	7.0 (4/57)
**70. Rahmani 2022**	23.3 (17/73)	30.3 (10/33)	17.5 (7/40)
**71. Rajwal 2004**	NR	NR	13.3 (4/30)
**72. Rohani 2021**	4.2 (5/120)	NR	NR
**73. Ronnblom 2015**	6.5 (6/92)	NR	NR
**74. Ruiz 2009**	8.5 (36/424)	NR	NR
**75. Rychwalski 1997**	12.5 (4/32)	16.7 (3/18)	7.1 (1/14)
**76. Sassine 2022**	NR	11.9 (78/654)	NR
**77. Schaefer 2018**	0.9 (151/17 587)	NR	NR
**78. Schoepfer 2022^b^**	NR	NR	NA
**79. Seo 1992^b^**	NA	NR	NR
**80. Shentova-Eneva 2019^b^**	NA	NA	NA
**81. Sladek 2014**	2.7 (16/585)	3.8 (14/368)	NR
**82. Sonavane 2018**	4.6 (3/65)	12.5 (3/24)	0 (0/41)
**83. Stenhammar 1994**	9.7 (3/31)	0 (0/9)	21.4 (3/14)
**84. Taskin 2023**	3.5 (2/57)	NR	NR
**85. Valentino 2015**	5.0 (15/300)	0.6 (1/163)	10.2 (14/137)
**86. Van Der Feen 2014**	2.0 (10/504)	NR	NR
**87. Yousif 2023**	NR	NR	NR
**88. Yu 2023**	1.6 (5/315)	1.4 (3/209)	0 (0/51)
**89. Zhou 2016^b^**	NA	NA	NA
**90. Zong 2019**	2.9 (2/70)	NR	NR

a The overall proportion of IM-EIMs could not be calculated as the manuscript only reported percentages and did not report proportions for EIMs.

b The overall proportion of IM-EIMs could not be calculated as the manuscripts reported that some patients experienced multiple EIMs, thus we were unable to add up the individual IM-EIMs to calculate the overall proportion of IM-EIMs.

Abbreviations: CD, Crohn’s disease; IBD, inflammatory bowel disease; IM-EIMs, immune-mediated extra-intestinal manifestations; NA, not available; NR, not reported; UC, ulcerative colitis.

**Table 3:** Prevalence and incidence of specific immune-mediated extra-intestinal manifestations in pediatric IBD, CD, and UC as reported by individual studies.

Study ID	IM-EIMs in IBD—% (*n*/*N*)	IM-EIMs in CD—% (*n*/*N*)	IM-EIMs in UC—% (*n*/*N*)
**1. Adamiak 2013**	NR	EN or PG: 2.6 PSC or AIH: 0.3	EN or PG: 1.2 PSC or AIH: 1.2
**2. Afarideh 2024**	EN: 3.3 (14/425) PG: 2.4 (10/425)	EN: 4.0 (11/276) PG: 2.5 (7/276)	EN: 2.0 (3/149) PG: 2.0 (3/149)
**3. Aguiar 2017**	Sacroiliitis: 31.2 (20/64)	NR	NR
**4. Al Saleem 2015**	NR	NR	Arthritis: 1.6 (3/188)
**5. Alexopoulou 2012**	PSC: 14.0 (8/57) AIH: 1.8 (1/57)	PSC: 12.2 (6/49) AIH: 2.0 (1/49)	PSC: 25.0 (2/8) AIH: 0 (0/8)
**6. Aloi 2013**	NR	NR	Axial arthropathies: 18.2 (20/110) Peripheral arthritis: 31.8 (35/110) PSC: 9.1 (10/110)
**7. Alreheili 2018**	Sacroiliitis: 3.2 (2/63) Arthritis: 11.1 (7/63) PSC: 7.9 (5/63) EN: 3.2 (2/63) PG: 1.6 (1/63) Uveitis/Episcleritis: 1.6 (1/63) Ankylosing Spondylitis: 3.2 (2/63)	Sacroiliitis: 5.6 (2/36) Arthritis: 16.7 (6/36) PSC: 2.8 (1/36) EN: 5.6 (2/36) PG: 2.8 (1/36) Uveitis/Episcleritis: 2.8 (1/36) Ankylosing Spondylitis: 5.6 (2/36)	Sacroiliitis: 0 (0/27) Arthritis: 3.7 (1/27) PSC: 14.8 (4/27) EN: 0 (0/27) PG: 0 (0/27) Uveitis/Episcleritis: 0 (0/27) Ankylosing Spondylitis: 0 (0/27)
**8. Arcucci 2022**	Peripheral arthritis: 7.0 (53/756) AIH: 8.6 (65/756)	Peripheral arthritis: 11.2 (28/250) AIH: 6.4 (16/250)	Peripheral arthritis: 4.9 (25/506) AIH: 9.7 (49/506)
**9. Ashton 2015**	EN: 1.2 (2/172) PSC: 1.2 (2/172) AIH: 0.6 (1/172)	EN: 0.9 (1/107) PSC: 0 (0/107) AIH: 0 (0/107)	EN: 2.0 (1/50) PSC: 4.0 (2/50) AIH: 2.0 (1/50)
**10. Ben Rabeh 2019**	Arthritis: 35.7 (5/14) PSC: 7.1 (1/14) Ankylosing spondylitis: 7.1 (1/14) EN: 7.1 (1/14)	NR	NR
**11. Bilgic Dagci 2022**	Peripheral arthritis: 36.0 (27/75) Axial arthritis: 20.0 (15/75) Enthesitis: 37.3 (28/75)	NR	NR
**12. Bramuzzo 2016**	PSC: 4.1 (28/677) AIH: 0.4 (3/677) Overlap syndrome: 2.2 (15/677)	NR	NR
**13. Cakir 2015**	PSC: 0.8 (1/127) Uveitis: 1.6 (2/127) EN: 1.6 (2/127)	PSC: 0 (0/29) Uveitis: 3.4 (1/29) EN: 3.4 (1/29)	PSC: 1.1 (1/90) Uveitis: 1.1 (1/90) EN: 1.1 (1/90)
**14. Cereser 2022**	NR	Sacroiliitis: 6.7 (2/30)	NR
**15. Chandrakumar 2019**	PSC: 5.8 (11/190) Incidence = 3.95 per 1000 person-years	NR	PSC: 10.0 (9/90) Incidence = 6.43 per 1000 person-years
**16. Cohen 2020**	Arthritis: 6.0 (6/100) Liver including unknown distribution of AIH, PSC, and overlap syndrome: 5.0 (5/100)	NR	NR
**17. Colletti 2019**	Incidence of psoriasis in TNF-α cohort: 0.06 events per 100 person-years Incidence of PSC in TNF-α cohort: 0.02 events per 100 person-years Incidence of lupus-like syndrome in TNF-α cohort: 0.02 events per 100 person-years Incidence of optic neuritis in TNF-α cohort: 0.01 events per 100 person-years Incidence of AIH in TNF-α cohort: 0.01 events per 100 person-years Incidence of PSC in non-biologic cohort: 0.02 events/100 person-years Incidence of AIH in non-biologic cohort: 0.01 events/100 person-years Incidence of arthritis in non-biologic cohort: 0.01 events/100 person-years	NR	NR
**18. Daniluk 2021**	PSC: 3.4 (4/119) ASC: 0.8 (1/119) AIH: 0.8 (1/119)	NR	PSC: 5.2 (4/77) ASC: 1.3 (1/77) AIH: 1.3 (1/77)
**19. Dass 2023**	EN: 6.0 (7/127)	NR	NR
**20. Deneau 2013**	PSC: 4.6 (28/607) AIH: 0.6 (2/607) ASC: 1.5 (9/607)	PSC: 0.6 (2/317) AIH: 0.3 (1/317) ASC: 0.9 (3/317)	PSC: 9.9 (26/262) AIH: 0.4 (1/262) ASC: 2.3 (6/262)
**21. Derfalvi 2022**	NR	Sacroiliitis: 1.2 (1/82) Enthesitis: 1.2 (1/82) Arthritis: 35.4 (29/82)	NR
**22. Dimakou 2015**	Arthritis: 5.0 (24/483) PSC: 1.2 (6/483) AIH: 0.6 (3/483) Overlap syndrome: 0.6 (3/483)	Arthritis: 9.6 (16/167) PSC: 1.8 (3/167) AIH: 0.6 (1/167) Overlap syndrome: 0.6 (1/167)	Arthritis: 1.9 (5/267) PSC: 0.7 (2/267) AIH: 0.7 (2/267) Overlap syndrome: 0.7 (2/267)
**23. Dong 2023**	NR	PSC: 1.0 (3/303)	NR
**24. Dotson 2010**	Ankylosing spondylitis: 0.4 (4/1009) Arthritis: 3.7 (37/1009) EN: 2.8 (28/1009) PG: 0.3 (3/1009) PSC: 1.5 (15/1009)	Ankylosing spondylitis: 0.5 (4/728) Arthritis: 4.4 (32/728) EN: 3.6 (26/728) PG: 0.4 (3/728) PSC: 1.0 (7/728)	Ankylosing spondylitis: 0 (0/281) Arthritis: 1.8 (5/281) EN: 0.7 (2/281) PG: 0 (0/281) PSC: 2.8 (8/281)
**25. Dzongowski 2023**	NR	NR	NR
**26. Fallahi 2009**	EN: 1.7 (1/59) PSC: 3.4 (2/59) AIH: 1.7 (1/59) Arthritis: 9.4 (6/59)	NR	NR
**27. Gerenli 2021**	Enthesitis: 45.2 (14/31)	Enthesitis: 54.5 (6/11)	Enthesitis: 41.2 (7/17)
**28. Gerenli 2022**	Sacroiliitis: 29.6 (8/27)	Sacroiliitis: 45.5 (5/11)	Sacroiliitis: 21.4 (3/14)
**29. Ghersin 2020**	Arthritis: 0.5 (12/2372) Uveitis: 0.08 (2/2372) PSC: 0.2 (5/2372) AIH: 0.7 (17/2372)	Arthritis: 0.6 (9/1612) Uveitis: 0.1 (2/1612) PSC: 0.1 (2/1612) AIH: 0.6 (9/1612)	Arthritis: 0.4 (3/760) Uveitis: 0 (0/760) PSC: 0.4 (3/760) AIH: 1.1 (8/760)
**30. Giani 2020**	Sacroiliitis: 14.7 (5/34)	Sacroiliitis: 12.5 (4/32)	Sacroiliitis: 50.0 (1/2)
**31. Goyal 2014**	PSC: 1.3 (21/1569) AIH: 0.1 (2/1569) Overlap syndrome: 0.4 (6/1569)	PSC: 0.7 (8/1075) AIH: 0 (0/1075) Overlap syndrome: 0.09 (1/1075)	PSC: 2.6 (13/494) AIH: 0.4 (2/494) Overlap syndrome: 1.0 (5/494)
**32. Greuter 2017**	Arthritis: 7.6 (25/329) Uveitis: 1.5 (5/329) EN: 1.5 (5/329) PG: 0.6 (2/329) PSC: 0.9 (3/329)	Arthritis: 11.0 (19/173) Uveitis: 2.9 (5/173) EN: 2.3 (4/173) PG: 0.6 (1/173) PSC: 0 (0/173)	Arthritis: 3.8 (6/156) Uveitis: 0 (0/156) EN: 0.6 (1/156) PG: 0.6 (1/156) PSC: 1.9 (3/156)
**33. Guariso 2010**	EN: 3.0 (4/133)	EN: 4.5 (3/67)	EN: 1.7 (1/58)
**34. Hlouskova 2019**	PSC: 2.9 (12/412) Overlap syndrome: 0.7 (3/412)	NR	NR
**35. Hofley 1993**	Uveitis: 4.1 (6/147)	Uveitis: 6.2 (6/97)	Uveitis: 0 (0/50)
**36. Horton 2012**	Arthritis: 2.3 (1/43) Enthesitis: 21.0 (9/43) EN: 4.6 (2/43)	NR	NR
**37.Isa 2018**	NR	Arthritis: 2.0 (1/51) EN: 3.9 (2/51) PG: 0 (0/51)	NR
**38. Ivkovic 2020**	Arthritis: 9.8 (5/51) EN: 3.9 (2/51) ASC: 2.0 (1/51) AIH: 3.9 (2/51)	Arthritis: 10.5 (2/19) EN: 5.3 (1/19) ASC: 0 (0/19) AIH: 0 (0/19)	Arthritis: 9.4 (3/32) EN: 3.1 (1/32) ASC: 3.1 (1/32) AIH: 6.3 (2/32)
**39. Jang 2021**	Ankylosing spondylitis: 0.6 (1/172) EN: 1.2 (2/172) PSC: 0.6 (1/172) AIH: 0.6 (1/172) PG: 0.6 (1/172) Uveitis: 0.6 (1/172)	Ankylosing spondylitis: 0.7 (1/137) EN: 0.7 (1/137) PSC: 0 (0/137) AIH: 0.7 (1/137) PG: 0.7 (1/137) Uveitis: 0 (0/137)	Ankylosing spondylitis: 0 (0/35) EN: 2.9 (1/35) PSC: 2.9 (1/35) AIH: 0 (0/35) PG: 0 (0/35) Uveitis: 2.9 (1/35)
**40. Jang 2022**	NR	NR	Arthritis: 1.0 (2/208) EN: 3.4 (7/208) PG: 1.0 (2/208) PSC: 5.8 (12/208)
**41. Jashmi 2022**	Arthritis: 2.7 (21/770) Axial arthritis: 1.2 (9/770) Peripheral arthritis: 1.6 (12/770)	NR	NR
**42. Jose 2009**	Arthritis: 4.3 (71/1649) Axial arthritis: 0.7 (12/1649) Peripheral arthritis: 2.7 (45/1649) Axial and peripheral arthritis: 0.8 (14/1649) Uveitis: 0.06 (1/1649) EN: 1.3 (21/1649) PG: 0.4 (6/1649) PSC: 1.5 (24/1649) AIH: 0.4 (6/1649)	NR	NR
**43. Kanavaki 2023**	PSC: 2.9 (2/70) AIH: 2.9 (2/70) Overlap syndrome: 2.9 (2/70)	NR	NR
**44. Kim 2017**	NR	NR	PSC: 5.5 (12/220)
**45. Klemenak 2024**	Arthritis: 2.3 (2/87) Uveitis: 3.4 (3/87) PSC: 3.4 (3/87)	NR	NR
**46. Kourti 2023**	NR	NR	NR
**47. Kwon 2022**	NR	NR	Sacroiliitis: 2.8 (4/142) Arthritis: 5.6 (8/142) Uveitis: 0.7 (1/142) PG: 1.4 (2/142) PSC: 0.7 (1/142)
**48. Lagercrantz 1958**	NR	NR	EN: 12.5 (6/48)
**49. Lee 2016**	NR	Arthritis: 2.7 (2/73) Uveitis: 1.4 (1/73) EN: 2.7 (2/73)	NR
**50. Lekovic 2011**	NR	NR	PG: 5.9 (1/17) AIH: 5.9 (1/17)
**51. Levy 2019**	Sacroiliitis: 1.1 (8/715) Arthritis: 3.2 (23/715)	NR	NR
**52. Lim 2013**	AIH: 0.6 (1/157) PSC: 3.8 (6/157) ASC: 0.6 (5/157)	NR	NR
**53. Lindsley 1974**	Ankylosing spondylitis: 3.7 (5/136) Arthritis: 13.2 (18/136)	Ankylosing spondylitis: 2.3 (2/86) Arthritis: 3.5 (3/86)	Ankylosing spondylitis: 6.0 (3/50) Arthritis: 30.0 (15/50)
**54. Malmborg 2016**	PSC: 6.8 (19/280) AIH: 0.4 (1/280) ASC: 0.7 (2/280)	NR	NR
**55. Maniscalco 2024**	Uveitis: 0.1 (6/4229)	Uveitis: 0.2 (4/1843)	Uveitis: 0.05 (1/2109)
**56. Mataly 2013**	NR	NR	Arthritis: 9.6 (11/115) PSC: 7.0 (8/115)
**57. Matar 2017**	Arthritis: 20.6 (36/175) PSC: 1.1 (2/175)	Arthritis: 20.9 (27/129) PSC: 0 (0/129)	Arthritis: 19.6 (9/46) PSC: 4.3 (2/46)
**58. Medynska-Przeczek 2023**	NR	PSC: 4.8 (1/21) AIH: 4.8 (1/21)	NR
**59. Merrick 2017**	Ankylosing spondylitis: 0.7 (6/809) PSC: 1.8 (15/809) Arthritis: 1.1 (9/809)	NR	NR
**60. Naviglio 2017**	Uveitis: 1.1 (1/94)	Uveitis: 2.2 (1/46)	Uveitis: 0 (0/46)
**61. Nemeth 1990**	PSC: 2.2 (1/46)	PSC: 0 (0/12)	PSC: 2.9 (1/34)
**62. Niewiem 2019**	Arthritis: 6.6 (19/287) EN: 2.4 (7/287) PSC: 5.6 (16/287) Overlap syndrome: 3.1 (9/287)	Arthritis: 10.0 (14/140) EN: 4.3 (6/140) PSC: 1.4 (2/140) Overlap syndrome: 0.7 (1/140)	Arthritis: 3.4 (5/147) EN: 0.7 (1/147) PSC: 9.5 (14/147) Overlap syndrome: 5.4 (8/147)
**63. Nir 2017**	Arthritis: 9.3 (40/430)	Arthritis: 12.3 (37/301)	Arthritis: 2.3 (3/129)
**64. Noble-Jamieson 2013**	NR	NR	PSC: 1.9 (1/52) Overlap syndrome: 30.8 (16/52)
**65. Ong 2014**	PSC: 4.1 (5/123)	PSC: 4.9 (4/82)	PSC: 3.6 (1/28)
**66. Ong 1994**	PSC: 3.1 (4/130)	PSC: 2.1 (1/47)	PSC: 3.6 (3/83)
**67. Ouldali 2018**	NR	Arthritis: 23.9 (65/272)	NR
**68. Passo 1986**	Arthritis: 12.7 (13/102)	Arthritis: 15.5 (9/58)	Arthritis: 9.1 (4/44)
**69. Pytrus 2011**	EN: 3.5 (5/143) PG: 1.4 (2/143)	EN: 1.2 (1/86) PG: 2.3 (2/86)	EN: 7.0 (4/57) PG: 0 (0/57)
**70. Rahmani 2022**	Arthritis: 16.4 (12/73) Ankylosing spondylitis: 1.4 (1/73) Uveitis: 1.4 (1/73) EN: 2.7 (2/73) PG: 1.4 (1/73)	Arthritis: 21.2 (7/33) Ankylosing spondylitis: 0 (0/33) Uveitis: 3.0 (1/33) EN: 6.1 (2/33) PG: 0 (0/33)	Arthritis: 12.5 (5/40) Ankylosing spondylitis: 2.5 (1/40) Uveitis: 0 (0/40) EN: 0 (0/40) PG: 2.5 (1/40)
**71. Rajwal 2004**	NR	NR	Arthritis: 3.3 (1/30) PSC: 10.0 (3/30)
**72. Rohani 2021**	PSC: 2.5 (3/120) AIH: 0.8 (1/120) ASC: 0.8 (1/120)	NR	NR
**73. Ronnblom 2015**	PSC: 3.3 (3/92) AIH: 3.3 (3/92)	NR	NR
**74. Ruiz 2009**	Arthritis: 8.5 (36/424)	NR	NR
**75. Rychwalski 1997**	Uveitis: 12.5 (4/32)	Uveitis: 16.7 (3/18)	Uveitis: 7.1 (1/14)
**76. Sassine 2022**	NR	Arthritis: 5.7 (37/654) Uveitis: 0.6 (4/654) EN: 3.7 (22/654) PG: 0 (0/654) PSC: 1.7 (11/654) AIH: 0.6 (4/654)	NR
**77. Schaefer 2018**	Uveitis: 0.9 (151/17 587)	NR	NR
**78. Schoepfer 2022**	NR	NR	Ankylosing spondylitis/sacroiliitis: 2.2 (4/184) Uveitis: 2.7 (5/184) EN: 2.2 (4/184) PG: 3.3 (6/184) PSC: 8.2 (15/184)
**79. Seo 1992**	Arthritis: 18.2 (4/22) EN: 4.5 (1/22) PG: 4.5 (1/22)	NR	NR
**80. Shentova-Eneva 2019**	Arthritis: 5.5 (5/91) PG: 1.1 (1/91)	NR	NR
**81. Sladek 2014**	PSC: 2.7 (16/585)	PSC: 3.8 (14/368)	NR
**82. Sonavane 2018**	Sacroiliitis: 3.1 (2/65) PG: 1.5 (1/65)	Sacroiliitis: 8.3 (2/24) PG: 4.2 (1/24)	Sacroiliitis: 0 (0/41) PG: 0 (0/41)
**83. Stenhammar 1994**	PSC: 9.7 (3/31)	PSC: 0 (0/9)	PSC: 21.4 (3/14)
**84. Taskin 2023**	Arthritis: 1.8 (2/57) EN: 1.8 (2/57)	NR	NR
**85. Valentino 2015**	PSC: 3.3 (10/300) AIH: 0 (0/300) ASC: 1.7 (5/300)	PSC: 0.6 (1/163) AIH: 0 (0/163) ASC: 0 (0/163)	PSC: 6.6 (9/137) AIH: 0 (0/137) ASC: 3.6 (5/137)
**86. Van Der Feen 2014**	ASC: 1.8 (9/504) AIH: 0.2 (1/504)	NR	NR
**87. Yousif 2023**	Arthritis: 1.9 (604/31 081) Uveitis: 0.2 (51/30 980) EN: 1.6 (509/31 796) PG: 0.9 (291/31 796)	NR	NR
**88. Yu 2023**	Uveitis: 1.6 (5/315)	Uveitis: 1.4 (3/209)	Uveitis: 0 (0/51)
**89. Zhou 2016**	Arthritis: 18.4 (9/49)	Arthritis: 19.5 (8/41)	Arthritis: 12.5 (1/8)
**90. Zong 2019**	Uveitis: 1.4 (1/70) EN: 1.4 (1/70)	NR	NR

Abbreviations: AIH, autoimmune hepatitis; ASC, autoimmune sclerosing cholangitis; CD, Crohn’s disease; EN, erythema nodosum; IBD, inflammatory bowel disease; IM-EIMs, immune-mediated extra-intestinal manifestations; NR, not reported; PG, pyoderma gangrenosum; PSC, primary sclerosing cholangitis; TNF-α, tumor necrosis factor-alpha; UC, ulcerative colitis.

The pooled prevalence of individual IM-EIMs in pediatric IBD is reported in Table 4. The musculoskeletal system was the most frequent IM-EIM. Enthesitis was the highest reported individual IM-EIM (23%, 95% CI, 4%-50%; I^2^ = 94.7%), followed by arthritis (8%, 95% CI, 6%-10%; I^2^ = 96%), sacroiliitis (8%, 95% CI, 3%-15%; I^2^ = 91%), and ankylosing spondylitis (2%, 95% CI, 1%-3%; I^2^ = 62%). IM-EIMs related to other systems were less common and included PSC (3%, 95% CI, 3%-4%; I^2^ = 84%), PSC-AIH overlap syndrome (3%, 95% CI, 1%-6%; I^2^ = 92%), and erythema nodosum (3%, 95% CI, 2%-3%; I^2^ = 57%). The lowest reported IM-EIMs in pediatric IBD at 1% or less included AIH, pyoderma gangrenosum, and uveitis.

**Table 4:** Pooled prevalence of individual immune-mediated extraintestinal manifestations by IBD type.

IM-EIM type	IBD type	Studies	Random effects (DerSimonian–Laird) pooled proportion	Heterogeneity (*I*^2^ statistic)
**Joint**				
**Ankylosing spondylitis**	IBD	8	0.02 (95% CI = 0.01 to 0.03)	62% (95% CI = 0% to 80.6%)
**Ankylosing spondylitis**	CD	5	0.01 (95% CI = 0.00 to 0.03)	41.2% (95% CI = 0% to 77.2%)
**Ankylosing spondylitis**	UC	6	0.02 (95% CI = 0.00 to 0.04)	65.9% (95% CI = 0% to 83.8%)
**Enthesitis**	IBD	4	0.23 (95% CI = 0.04 to 0.50)	94.7% (95% CI = 90.3% to 96.6%)
**Enthesitis**	CD	2	0.20 (95% CI = 0.04 to 0.83)	94.9% (95% CI = NA)
**Enthesitis**	UC	1	0.42 (95% CI = 0.20 to 0.65)	NA
**Sacroiliitis**	IBD	9	0.08 (95% CI = 0.03 to 0.15)	90.7% (95% CI = 85.1% to 93.6%)
**Sacroiliitis**	CD	6	0.10 (95% CI = 0.04 to 0.20)	72.7% (95% CI = 11.4% to 86.3%)
**Sacroiliitis**	UC	5	0.05 (95% CI = 0.01 to 0.13)	67.4% (95% CI = 0% to 85.3%)
**Arthritis**	IBD	40	0.08 (95% CI = 0.06 to 0.10)	96% (95% CI = 95.5% to 96.5%)
**Arthritis**	CD	19	0.11 (95% CI = 0.07 to 0.16)	95.6% (95% CI = 94.7% to 96.4%)
**Arthritis**	UC	20	0.07 (95% CI = 0.04 to 0.10)	90.7% (95% CI = 87.5% to 92.7%)
**Liver**				
**AIH**	IBD	22	0.01 (95% CI = 0.01 to 0.02)	88.2% (95% CI = 83.9% to 90.9%)
**AIH**	CD	13	0.01 (95% CI = 0.00 to 0.02)	78.7% (95% CI = 61.8% to 86.2%)
**AIH**	UC	12	0.02 (95% CI = 0.01 to 0.04)	88.2% (95% CI = 81.5% to 91.7%)
**PSC**	IBD	41	0.03 (95% CI = 0.03 to 0.04)	84.5% (95% CI = 80% to 87.5%)
**PSC**	CD	23	0.01 (95% CI = 0.01 to 0.02)	71% (95% CI = 53.2% to 80%)
**PSC**	UC	28	0.05 (95% CI = 0.04 to 0.07)	81.3% (95% CI = 73.6% to 85.9%)
**Overlap syndrome**	IBD	8	0.03 (95% CI = 0.01 to 0.06)	91.6% (95% CI = 86.4% to 94.2%)
**Overlap syndrome**	CD	3	0.0041 (95% CI = 0.00 to 0.01)	43.1% (95% CI = 0% to 83%)
**Overlap syndrome**	UC	4	0.06 (95% CI = 0.01 to 0.15)	94.3% (95% CI = 89.2% to 96.4%)
**Skin**				
**Erythema nodosum**	IBD	27	0.03 (95% CI = 0.02 to 0.03)	57.4% (95% CI = 28.9% to 71.3%)
**Erythema nodosum**	CD	15	0.03 (95% CI = 0.03 to 0.04)	0% (95% CI = 0% to 46.4%)
**Erythema nodosum**	UC	15	0.02 (95% CI = 0.01 to 0.04)	42% (95% CI = 0% to 67.2%)
**P. gangrenosum**	IBD	18	0.01 (95% CI = 0.01 to 0.01)	65.7% (95% CI = 37.5% to 78%)
**P. gangrenosum**	CD	10	0.01 (95% CI = 0.00 to 0.02)	65.2% (95% CI = 14.8% to 80.6%)
**P. gangrenosum**	UC	12	0.01 (95% CI = 0.01 to 0.02)	36.4% (95% CI = 0% to 66.6%)
**Eye**				
**Uveitis**	IBD	20	0.01 (95% CI = 0.01 to 0.01)	91.1% (95% CI = 88.2% to 93%)
**Uveitis**	CD	13	0.02 (95% CI = 0.01 to 0.03)	79% (95% CI = 62.4% to 86.3%)
**Uveitis**	UC	13	0.01 (95% CI = 0.00 to 0.01)	60.2% (95% CI = 12.2% to 76.9%)

Abbreviations: AIH, autoimmune hepatitis; CD, Crohn’s disease; CI, confidence interval; IBD, inflammatory bowel disease; IM-EIM, immune-mediated extraintestinal manifestations; P., pyoderma; PSC, primary sclerosing cholangitis; UC, ulcerative colitis.

#### Prevalence of IM-EIMs in pediatric CD

**Figure 2:**
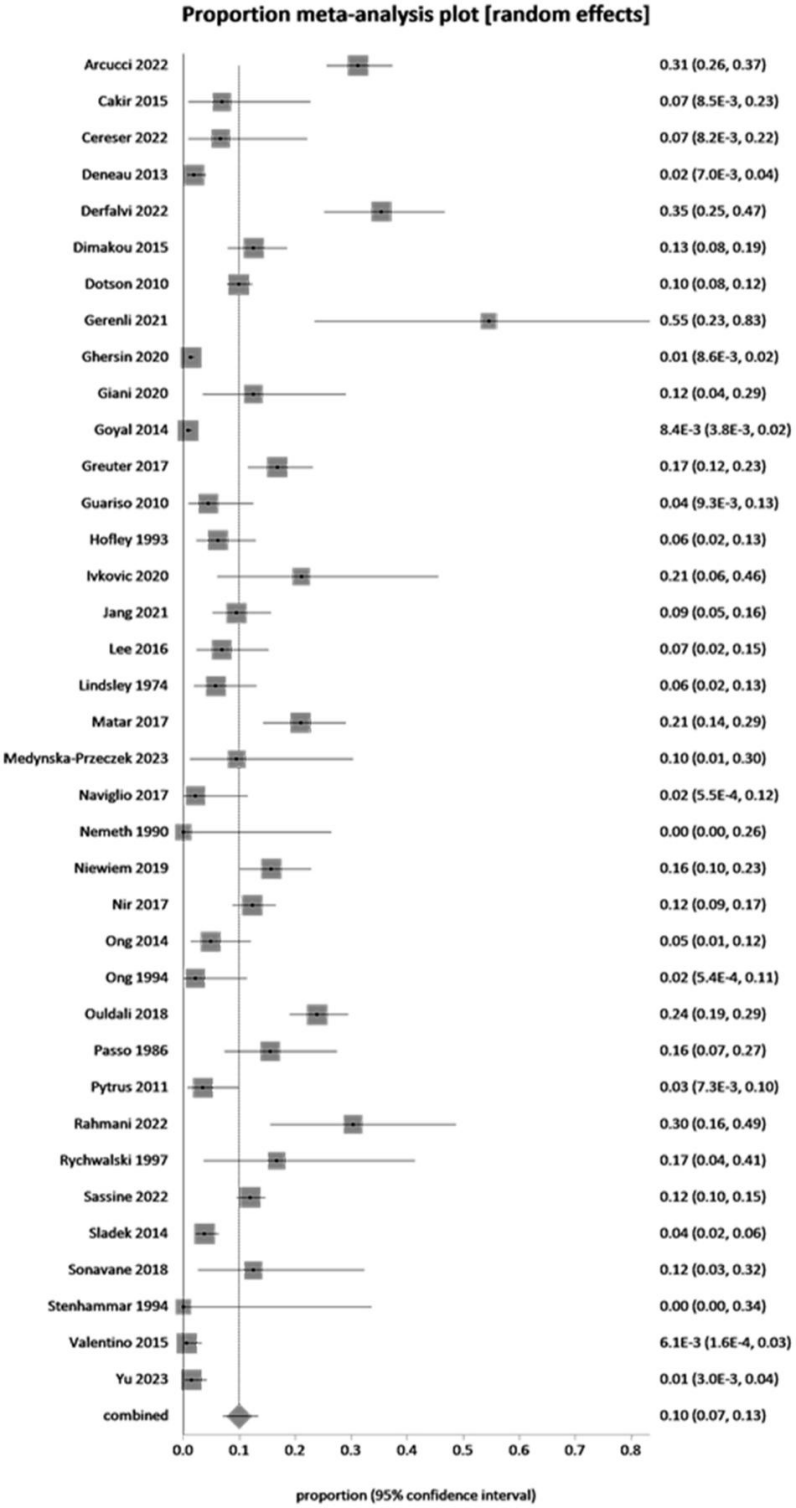
Pooled overall immune-mediated extra-intestinal manifestations in pediatric Crohn’s disease.

The cumulative prevalence of IM-EIMs in pediatric CD as reported by the individual studies is shown in Table 2. The prevalence of IM-EIMs in pediatric CD ranged from a low of 0% to a high of 54.5% (6/11). The overall pooled prevalence of IM-EIMs in pediatric CD across 37 eligible studies including 7657 patients was 10% (95% CI, 7%-13%, I^2^ = 94.8%; Figure 2). The prevalence of individual IM-EIMs in pediatric CD as reported by the individual studies is shown in Table 3. The pooled prevalence of individual IM-EIMs in pediatric CD is reported in Table 4. Enthesitis was the highest reported individual IM-EIM (20%, 95% CI, 4%-83%; I^2^ = 95%), followed by arthritis (11%, 95% CI, 7%-16%; I^2^ = 96%), sacroiliitis (10%, 95% CI, 4%-20%; I^2^ = 73%), erythema nodosum (3%, 95% CI, 3%-4%; I^2^ = 0%), and uveitis (2%, 95% CI, 1%-3%; I^2^ = 79%). Ankylosing spondylitis, AIH, PSC, PSC-AIH overlap syndrome, and pyoderma gangrenosum were the lowest reported IM-EIMs in pediatric CD at 1% or less.

#### Prevalence of IM-EIMs in pediatric UC

**Figure 3:**
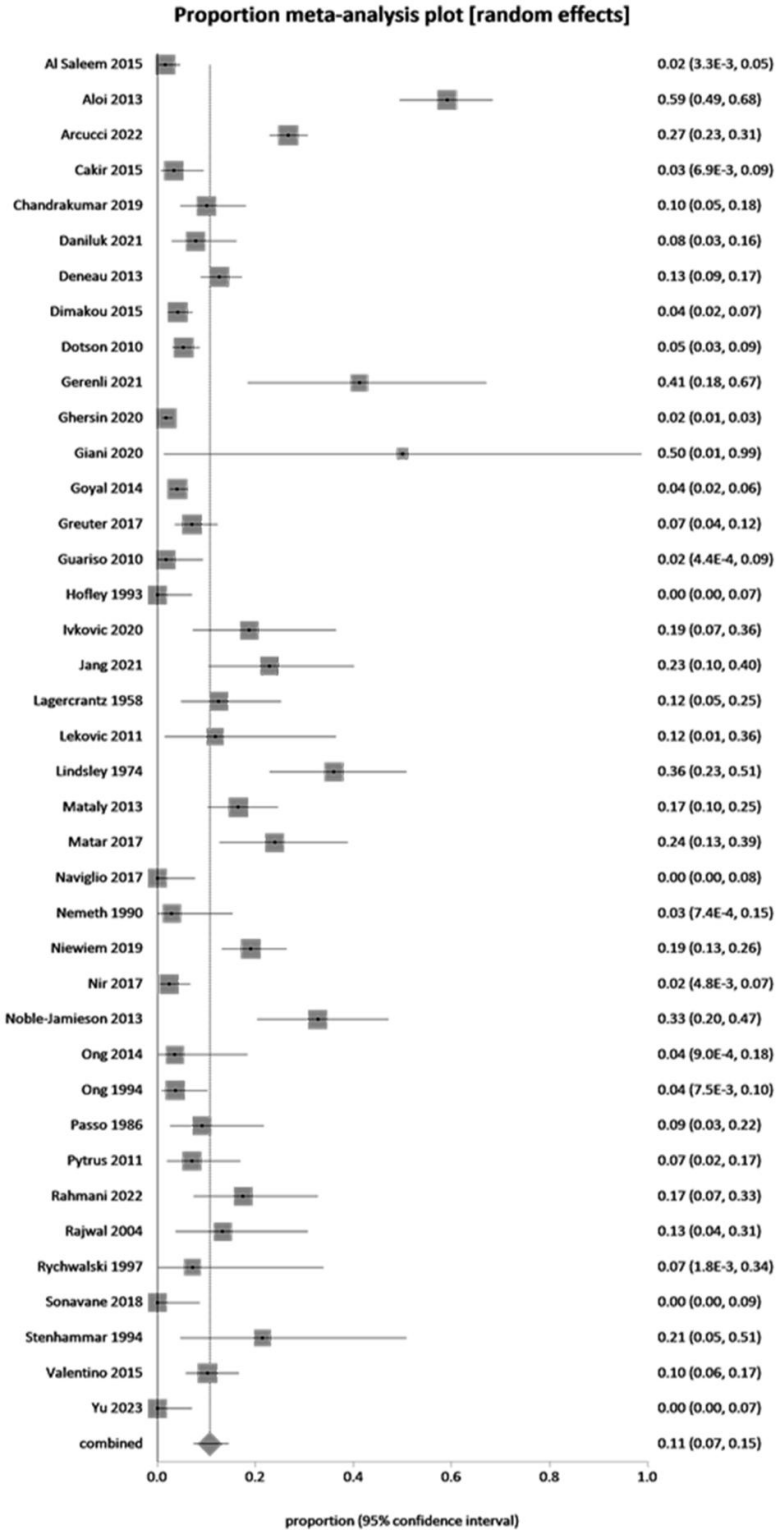
Pooled overall immune-mediated extra-intestinal manifestations in pediatric ulcerative colitis.

The cumulative prevalence of IM-EIMs in pediatric UC as reported by the individual studies is shown in Table 2. The prevalence of IM-EIMs in pediatric UC ranged from a low of 0% to a high of 59.1% (65/110). The overall pooled prevalence of IM-EIMs in pediatric UC across 39 eligible studies including 4698 patients was 11% (95% CI, 7%-15%, I^2^ = 93.3%; Figure 3). The prevalence of individual IM-EIMs in pediatric UC as reported by the individual studies is shown in Table 3. The pooled prevalence of individual IM-EIMs in pediatric UC is reported in Table 4. Enthesitis was the highest reported individual IM-EIM (42%, 95% CI, 20%-65%; 1 study) in pediatric UC. Arthritis (7%, 95% CI, 4%-10%; I^2^ = 91%) and PSC-AIH overlap syndrome (6%, 95% CI, 1%-15%; I^2^ = 94%) were the second and third highest reported individual IM-EIMs in pediatric UC, respectively, followed by sacroiliitis (5%, 95% CI, 1%-13%; I^2^ = 67%), PSC (5%, 95% CI, 4%-7%; I^2^ = 81%), ankylosing spondylitis (2%, 95% CI, 0%-4%; I^2^ = 66%), AIH (2%, 95% CI, 1%-4%; I^2^ = 88%), and erythema nodosum (2%, 95% CI, 1%-4%; I^2^ = 42%). Pyoderma gangrenosum and uveitis were the lowest reported IM-EIMs in pediatric UC at 1% or less.

The largest differences in the prevalence of IM-EIMs stratified by IBD types included PSC, which was 4% higher in UC than CD, arthritis which was 4% higher in CD than UC, and sacroiliitis which was 5% higher in CD than UC.

## Discussion

This systematic review and meta-analysis highlight the incidence and prevalence of IM-EIMs in pediatric IBD (pIBD). Our analysis identified that up to half of children with IBD may experience at least one IM-EIM, a proportion similar to that reported in the adult IBD population.^3^^,^^21^ Early recognition of IM-EIMs is important for the management of IBD to prevent further comorbidity, help select therapeutic strategies and influence overall treatment outcomes.

Musculoskeletal IM-EIMs had the highest pooled prevalence with enthesitis having the highest individual pooled prevalence for both CD and UC. While the pooled prevalence of enthesitis might be inflated by the low number of studies that reported this IM-EIM, musculoskeletal IM-EIMs among adult studies are also reported frequently with the prevalence of enthesitis as high as 54%.^21^

The second most prevalent group was hepatic IM-EIMs including PSC and PSC-AIH overlap syndrome in UC with rates ranging from 5% to 6%. Consistent with the adult literature, the pooled prevalence of PSC in UC was higher than in CD (6% vs 1%, respectively).^21^^,^^22^ PSC-AIH overlap syndrome had a similar pooled prevalence compared to PSC alone. Historically, PSC-AIH overlap syndrome was first described in the pediatric population and was thought of as a more common pediatric entity. However, more recently some experts argue that PSC-AIH overlap syndrome may be a continuum of PSC that presents with more inflammatory features earlier in the disease course of younger individuals.^23^^,^^24^

Skin and ocular manifestations were the least prevalent IM-EIMs reported in the pediatric literature. This is in contrast with the adult literature where those organ systems are the most commonly reported IM-EIMs.^25^ It is unclear if this truly represents a lower prevalence in younger patients, or if these IM-EIMs were relatively underreported in pIBD studies.

We sought to include the incidence of each IM-EIM in this systematic review. However, as incidence statistics require the numbers of IM-EIMs over a specific time period, studies that reported on the incidence of individual IM-EIMs were scarce and were best documented for PSC.

Our systematic review focused on the incidence and prevalence of IM-EIMS in pIBD and did not focus on the treatment of IM-EIMs in pIBD. Thus, a further systematic review focusing on the treatment of IM-EIMS in pIBD is needed.

It is crucial for pIBD studies to evaluate treatments not only for intestinal manifestations, but also for common IM-EIMs, to improve multisystemic outcomes. To further address this issue, the International Organization for the study of Inflammatory Bowel Disease (IOIBD) developed the Endpoints for extraintestinal manifestations in IBD trials: the EXTRA consensus.^12^ While the IOIBD paper focused on EIM consensus recommendations in IBD trials, further pediatric-focused EIM endpoint consensus is needed to systematically recognize, document and treat IM-EIMs. While pediatric Centers that are affiliated with the ImproveCareNow Learning Health System systematically document IM-EIMs at each clinic visit, global pediatric IM-EIM recommendations could enhance the literature around epidemiology and management of IM-EIMs beyond organizations such as ImproveCareNow.^26^

A limitation of this systematic review was that the definition of IM-EIMs was stringent and only focused on children who had an established diagnosis of IBD. While this may have excluded some patients who had IM-EIMs presented prior to IBD diagnosis, this was an intentional effort to capture the true epidemiology of IM-EIMs among pIBD patients. Furthermore, as there are various definitions for IM-EIMs, we included liver, musculoskeletal, dermatological and ophthalmological manifestations based on expert consensus between the authors. This consensus only included IM-EIMs with objective findings, and subjective symptoms such as arthralgia were excluded. Lastly, as this was a pediatric meta-analysis, the majority of primary studies were small convenience samples and were not primarily designed to report on the epidemiology of IM-EIMs. Thus, this contributed to the significant statistical heterogeneity in our meta-analysis.

In conclusion, this systematic review and meta-analysis found that at least one IM-EIM occurs in almost half of patients with pIBD. The pooled prevalence of individual IM-EIMs ranged from 1% to 23%. As epidemiologic studies of pediatric EIMs are rare, the IBD community needs to focus on describing EIMs in a robust standardized fashion. The combination of biological linkage studies,^27^ and further standardization of IM-EIMs endpoints in trials will improve multi-systemic outcomes in pIBD patients.

## Supplementary Material

otag024_Supplementary_Data

## Data Availability

The data underlying this article will be shared on reasonable request to the corresponding author.
