# Supplementary material for: Incidence and prevalence of immune-mediated extraintestinal manifestations in pediatric inflammatory bowel disease: a systematic review and meta-analysis
Source: Crohns Colitis 360. 2026 Mar 31;8(2):otag024. doi: 10.1093/crocol/otag024 (PMC13099386; doi:10.1093/crocol/otag024)
Supplement: otag024_Supplementary_Data [file otag024_supplementary_data.docx]

**Supplementary material for:**

**Incidence and prevalence of immune-mediated extraintestinal manifestations in paediatric inflammatory bowel disease: a systematic review and meta-analysis**

Table of Contents

[Supplementary Appendix 1: Search strategy 2](#_Toc197527750)

[Supplementary Figure 1: PRISMA Flow Diagram 7](#_Toc197527751)

[Supplementary Table 1: Excluded studies 8](#_Toc197527752)

[Supplementary Table 2: Characteristics of included studies. 15](#_Toc197527753)

[Supplementary Table 3: Quality assessment of cohort and cross-sectional studies (Newcastle-Ottawa scale) 30](#_Toc197527754)

[References 34](#_Toc197527755)

# Supplementary Appendix 1: Search strategy

**MEDLINE**

1. Inflammatory bowel disease.mp. or IBD.mp. or exp Inflammatory bowel disease/

2. exp Crohn disease/ or crohn*.mp. or regional ileitis.mp.

3. ulcerative colitis.mp. or colitis.mp. or exp Colitis, Ulcerative/

4. (proctocolitis or proctosigmoiditis or rectocolitis or rectosigmoiditis or proctitis or distal colitis).mp.

5. Early ADJ onset ADJ inflammatory ADJ bowel ADJ disease*.ti. or Early ADJ onset ADJ inflammatory ADJ bowel ADJ disease*.ab.

6. pediatric Inflammatory bowel disease.ti. or pediatric Inflammatory bowel disease.ab. or paediatric Inflammatory bowel disease.ti. or paediatric Inflammatory bowel disease.ab. or pIBD.ti. or pIBD.ab.

7. or/1-6

8. children.ti. or children.ab. or Exp Children/

9. adolescent.ti. or adolescent.ab. or Exp Adolescent/

10. child.ti. or child.ab. or Exp child/

11. Pediatric*.mp.

12. Paediatric*.mp.

13. or/ 8-12

14. Musculoskeletal* manifestation*.mp.

15. Axial arthropathy*.mp.

16. Peripheral arthropathy*.mp.

17. Sacroiliitis.mp. or exp Sacroiliitis/

18. ankylosing spondylitis*.mp. or exp Spondylitis, Ankylosing/

19. enthesitis*.mp

20. arthritis*.mp.

21. Ophthal* manifestation*.mp.

22. episcleritis*.mp.

23. uveitis*.mp.

24. scleritis*.mp.

25. Derm* manifestation*.mp.

26. Erythema nodosum.mp. or exp Erythema Nodosum/

27. Pyoderma gangrenosum.mp. or exp Pyoderma Gangrenosum/

28. Sweet syndrome.mp. or exp Sweet Syndrome/

29. acute febrile neutrophilic dermatosis.mp.

30. Granulomatous cutaneous*.mp.

31. Liver manifestation*.mp.

32. Hepatic* manifestation*.mp.

33. Primary sclerosing cholangitis.mp. or exp Cholangitis, Sclerosing/

34. Autoimmune hepatitis.mp. or exp Hepatitis, Autoimmune/

35. Granulomatous hepatitis.mp.

36. or/14-35

37. 7 and 13 and 36

38. Limit 37 to dt=20230606-20240506

**Embase**

1. Inflammatory bowel disease.mp. or IBD.mp. or exp Inflammatory bowel disease/

2. exp Crohn disease/ or crohn*.mp. or regional ileitis.mp.

3. ulcerative colitis.mp. or colitis.mp. or exp Colitis, Ulcerative/

4. (proctocolitis or proctosigmoiditis or rectocolitis or rectosigmoiditis or proctitis or “distal colitis”).mp.

5. Early ADJ onset ADJ inflammatory ADJ bowel ADJ disease*.ti. or Early ADJ onset ADJ inflammatory ADJ bowel ADJ disease*.ab.

6. pediatric Inflammatory bowel disease.ti. or pediatric Inflammatory bowel disease.ab. or paediatric Inflammatory bowel disease.ti. or paediatric Inflammatory bowel disease.ab. or pIBD.ti. or pIBD.ab.

7. or/1-6

8. children.ti. or children.ab. or Exp Children/

9. adolescent.ti. or adolescent.ab. or Exp Adolescent/

10. child.ti. or child.ab. or Exp child/

11. Pediatric*.mp.

12. Paediatric*.mp.

13. or/ 8-12

14. Musculoskeletal* manifestation*.mp.

15. Axial arthropathy*.mp.

16. Peripheral arthropathy*.mp.

17. Sacroiliitis.mp. or exp Sacroiliitis/

18. ankylosing spondylitis*.mp. or exp Spondylitis, Ankylosing/

19. enthesitis*.mp

20. arthritis*.mp.

21. Ophthal* manifestation*.mp.

22. episcleritis*.mp.

23. uveitis*.mp.

24. scleritis*.mp.

25. Derm* manifestation*.mp.

26. Erythema nodosum.mp. or exp Erythema Nodosum/

27. Pyoderma gangrenosum.mp. or exp Pyoderma Gangrenosum/

28. Sweet syndrome.mp. or exp Sweet Syndrome/

29. acute febrile neutrophilic dermatosis.mp.

30. Granulomatous cutaneous*.mp.

31. Liver manifestation*.mp.

32. Hepatic* manifestation*.mp.

33. Primary sclerosing cholangitis.mp. or exp Cholangitis, Sclerosing/

34. Autoimmune hepatitis.mp. or exp Hepatitis, Autoimmune/

35. Granulomatous hepatitis.mp.

36. or/14-35

37. 7 and 13 and 36

38. Limit 37 to dc=20230606-20240506

**Cochrane CENTRAL**

#1. MeSH descriptor: [Inflammatory Bowel Diseases] explode all trees

#2. MeSH descriptor: [Colitis, Ulcerative] explode all trees

#3. MeSH descriptor: [Crohn disease] explode all trees

#4. Inflammatory bowel disease or IBD or crohn or regional ileitis or ulcerative colitis or proctocolitis or proctosigmoiditis or rectocolitis or rectosigmoiditis or proctitis or distal colitis or early onset inflammatory bowel disease* or pediatric Inflammatory bowel disease or pediatric Inflammatory bowel disease or paediatric Inflammatory bowel disease or pIBD

#5. #1 or #2 or #3 or #4

#6. MeSH descriptor: [Child] explode all trees

#7. MeSH descriptor: [Adolescent] explode all trees

#8. children or adolescent or child or Pediatric* or Paediatric

#9. #6 or #7 or #8

#10. Musculoskeletal* manifestation* or Axial arthropathy* or Peripheral arthropathy* or Sacroiliitis or ankylosing spondylitis* or enthesitis* or arthritis or Ophthal* manifestation* or episcleritis* or uveitis* or scleritis* or Derm* manifestation* or Erythema nodosum or Pyoderma gangrenosum or Sweet syndrome or acute febrile neutrophilic dermatosis or Granulomatous cutaneous* or Liver manifestation* or Hepatic* manifestation or Primary sclerosing cholangitis or Autoimmune hepatitis or Granulomatous hepatitis

#11. #5 and #9 and #10

**Clinical trials.gov**

1. Pediatric and inflammatory bowel diseases

2. Paediatric and inflammatory bowel diseases

3. Pediatric and Crohn

4. Paediatric and Crohn

5. Pediatric and ulcerative colitis

6. Paediatric and ulcerative colitis

7. Child and inflammatory bowel diseases

8. Child and Crohn

9. Child and ulcerative colitis

10. Adolescent and inflammatory bowel diseases

11. Adolescent and Crohn

12. Adolescent and ulcerative colitis

# Supplementary Figure 1: PRISMA Flow Diagram

Additional records identified through other sources
(n = 3)

Records after duplicates removed
(n = 6,167)

Records identified through database searching
(n = 9,323)

Full-text articles excluded, with reasons
(n = 225)

- 103 No rates between 2‑17 years old
- 3 EIM not verified/ preclinical
- 7 less than 10 subjects
- 4 EIM not of interest
- 10 Not English language
- 5 Not original research article
- 29 Data overlaps with other record
- 61 Inadequate information to calculate primary outcome
- 1 convenience sample
- 2 unclear diagnosis

## Screening

## Eligibility

## Identification

## Included

Records excluded
(n = 5,851)

Records screened
(n =6,167)

Full-text articles assessed for eligibility
(n =316)

Studies included in qualitative synthesis
(n = 91 reports of 90 studies)

Studies included in quantitative synthesis (meta-analysis)
**(n = 91)**

# Supplementary Table 1: Excluded studies

| **Number** | **Reference** | **Reason for exclusion** |
| --- | --- | --- |
| 1 | Aadland, et al. 1987^1^ | No rates between 2-17yo |
| 2 | Abbasian, et al. 2012^2^ | No rates between 2-17yo |
| 3 | Adeniyi, et al. 2020^3^ | Less than 10 patients |
| 4 | Aguilera et al 2023^4^ | Unable to calculate incidence/prevalence |
| 5 | Al-Mendalawi, 2018^5^ | Not original research article |
| 6 | Al-Mendalawi, 2019^6^ | Not original research article |
| 7 | Aletaha, et al. 2019^7^ | No rates between 2-17yo |
| 8 | Alkhouri, et al. 2009^8^ | Less than 10 patients |
| 9 | Almaas, 2019^9^ | Not original research article |
| 10 | Alreheili, et al. 2016^10^ | Data overlaps with other record |
| 11 | Amouri, et al. 2016^11^ | No rates between 2-17yo |
| 12 | Araujo, et al. 2022^12^ | Unable to calculate incidence/prevalence |
| 13 | Assa, et al. 2017^13^ | Data overlaps with other record |
| 14 | Assa, et al. 2018^14^ | Data overlaps with other record |
| 15 | Aziz, et al. 2017^15^ | Unable to calculate incidence/prevalence |
| 16 | Bandinelli, et al. 2014^16^ | EIM not verified/preclinical |
| 17 | Barreiro-De Acosta, et al. 2007^17^ | No rates between 2-17yo |
| 18 | Barrie, et al. 2013^18^ | No rates between 2-17yo |
| 19 | Batticciotto, et al. 2014^19^ | EIM not verified/preclinical |
| 20 | Batticciotto, et al. 2015^20^ | EIM not verified/preclinical |
| 21 | Beauvais, et al. 1995^21^ | Not English language |
| 22 | Ben Abdesslem, et al. 2019^22^ | Not English language |
| 23 | Bennett, et al. 1991^23^ | Unable to calculate incidence/prevalence |
| 24 | Biedermann, et al. 2019^24^ | No rates between 2-17yo |
| 25 | Boberg, et al. 1994^25^ | No rates between 2-17yo |
| 26 | Bornman, et al. 1993^26^ | No rates between 2-17yo |
| 27 | Boskovic, et al. 2011^27^ | Unable to calculate incidence/prevalence |
| 28 | Bracci, et al. 2013^28^ | Unable to calculate incidence/prevalence |
| 29 | Brakenhoff, et al. 2014^29^ | No rates between 2-17yo |
| 30 | Bramuzzo, et al. 2014^30^ | Data overlaps with other record |
| 31 | Bramuzzo, et al. 2021^31^ | Unable to calculate incidence/prevalence |
| 32 | Broome, et al. 1994^32^ | No rates between 2-17yo |
| 33 | Bruining, et al. 2008^33^ | No rates between 2-17yo |
| 34 | Burgos, et al. 2019^34^ | Unable to calculate incidence/prevalence |
| 35 | Card, et al. 2016^35^ | No rates between 2-17yo |
| 36 | Cardile, et al. 2015^36^ | Unable to calculate incidence/prevalence |
| 37 | Cardile, et al. 2019^37^ | Unable to calculate incidence/prevalence |
| 38 | Cardile, et al. 2012^38^ | EIM not of interest |
| 39 | Caspersen, et al. 2008^39^ | No rates between 2-17yo |
| 40 | Catassi et al 2023a^40^ | Unable to calculate incidence/prevalence |
| 41 | Catassi et al 2023b^41^ | Unable to calculate incidence/prevalence |
| 42 | Chan, et al. 2018^42^ | No rates between 2-17yo |
| 43 | Chapuy, et al. 2020^43^ | Unable to calculate incidence/prevalence |
| 44 | Cheikhna, et al. 2019^44^ | Unable to calculate incidence/prevalence |
| 45 | Chew, et al. 2019^45^ | Unable to calculate incidence/prevalence |
| 46 | Chlebowczyk, et al. 2012^46^ | Unable to calculate incidence/prevalence |
| 47 | Conti, et al. 2005^47^ | Unable to calculate incidence/prevalence |
| 48 | Cordes, et al. 2019^48^ | No rates between 2-17yo |
| 49 | Coutzac, et al. 2015^49^ | No rates between 2-17yo |
| 50 | Csizmadia, et al. 2019^50^ | Unable to calculate incidence/prevalence |
| 51 | Daum, et al. 1979^51^ | Unclear if IBD |
| 52 | Davis, et al. 1978^52^ | No rates between 2-17yo |
| 53 | Demir et al 2023^53^ | Unable to calculate incidence/prevalence |
| 54 | Derfalvi, et al. 2014^54^ | Data overlaps with other record |
| 55 | Deshayes, et al. 1976^55^ | Not English language |
| 56 | Dilillo, et al. 2014^56^ | Unclear if IBD |
| 57 | Dupre, et al. 2020^57^ | No rates between 2-17yo |
| 58 | El-Shabrawi, et al. 2020^58^ | Convenience sample |
| 59 | Ernst, et al. 1991^59^ | No rates between 2-17yo |
| 60 | Farahmand, et al. 2019^60^ | Unable to calculate incidence/prevalence |
| 61 | Farinelli, et al. 2017^61^ | No rates between 2-17yo |
| 62 | Fedor, et al. 2021^62^ | No rates between 2-17yo |
| 63 | Felekis, et al. 2009^63^ | No rates between 2-17yo |
| 64 | Feler, et al. 2018^64^ | Data overlaps with other record |
| 65 | Fraga, et al. 2017^65^ | No rates between 2-17yo |
| 66 | Fumery, et al. 2019^66^ | Unable to calculate incidence/prevalence |
| 67 | Gerenli, et al. 2019^67^ | Data overlaps with other record |
| 68 | Ghersin, et al. 2018^68^ | Data overlaps with other record |
| 69 | Ghione, et al. 2012^69^ | Unable to calculate incidence/prevalence |
| 70 | Giani, et al. 2019a^70^ | Unable to calculate incidence/prevalence |
| 71 | Giani, et al. 2019b^71^ | Unable to calculate incidence/prevalence |
| 72 | Gilat, et al. 1976^72^ | Unable to calculate incidence/prevalence |
| 73 | Gomollon, et al. 2022^73^ | No rates between 2-17yo |
| 74 | Goyal, et al. 2012^74^ | Data overlaps with other record |
| 75 | Goyal et al 2016^75^ | Less than 10 patients |
| 76 | Greuter, et al. 2016^76^ | Data overlaps with other record |
| 77 | Grzybowska-Chlebowczyk, et al. 2008^77^ | Unable to calculate incidence/prevalence |
| 78 | Gupta, et al. 2007^78^ | Unable to calculate incidence/prevalence |
| 79 | Gutierrez, et al. 2022^79^ | No rates between 2-17yo |
| 80 | Haapamaki, et al. 2011^80^ | No rates between 2-17yo |
| 81 | Hammoudeh, et al. 2017^81^ | No rates between 2-17yo |
| 82 | Hattar, et al. 2012^82^ | Unable to calculate incidence/prevalence |
| 83 | Hiller, et al. 2019^83^ | No rates between 2-17yo |
| 84 | Hirche, et al. 2004^84^ | No rates between 2-17yo |
| 85 | Holowach, et al. 1956^85^ | Unable to calculate incidence/prevalence |
| 86 | Hopkins, et al. 1974^86^ | Less than 10 patients |
| 87 | Hsu, et al. 2017^87^ | No rates between 2-17yo |
| 88 | Jacobs, et al. 1977^88^ | No rates between 2-17yo |
| 89 | Jalan, et al. 1970^89^ | No rates between 2-17yo |
| 90 | Jang et al. 2017^90^ | Data overlaps with other record |
| 91 | Jang, et al. 2018^91^ | Data overlaps with other record |
| 92 | Jangi, et al. 2020^92^ | No rates between 2-17yo |
| 93 | Jiang, 2021^93^ | Not original research article |
| 94 | Jolving et al. 2024^94^ | Unable to calculate incidence/prevalence |
| 95 | Jose, et al. 2009^95^ | Data overlaps with other record |
| 96 | Kalubowila, et al. 2018^96^ | No rates between 2-17yo |
| 97 | Kappelman, et al. 2009^97^ | Data overlaps with other record |
| 98 | Kappelman, et al. 2011^98^ | EIM not of interest |
| 99 | Kelly, et al. 1997^99^ | No rates between 2-17yo |
| 100 | Kumagai, et al. 2018^100^ | No rates between 2-17yo |
| 101 | Lakatos, et al. 2003^101^ | No rates between 2-17yo |
| 102 | Lam, et al. 2018^102^ | Unable to calculate incidence/prevalence |
| 103 | Lazdr, et al. 2017^103^ | No rates between 2-17yo |
| 104 | Ledder, et al. 2010^104^ | No rates between 2-17yo |
| 105 | Lee, 2016^105^ | No rates between 2-17yo |
| 106 | Lepisto, et al. 2008^106^ | No rates between 2-17yo |
| 107 | Lev-Tzion, et al. 2018^107^ | No rates between 2-17yo |
| 108 | Levitt, et al. 1991^108^ | No rates between 2-17yo |
| 109 | Lopez, et al. 2018^109^ | Unable to calculate incidence/prevalence |
| 110 | Louis, et al. 2016^110^ | Data overlaps with other record |
| 111 | Louis, et al. 2018^111^ | No rates between 2-17yo |
| 112 | Ludvigsson, et al. 2017^112^ | Unable to calculate incidence/prevalence |
| 113 | Lustosa et al. 2017^113^ | Less than 10 patients |
| 114 | Malaty, et al. 2011a^114^ | Unable to calculate incidence/prevalence |
| 115 | Malaty, et al. 2011b^115^ | Unable to calculate incidence/prevalence |
| 116 | Malaty, et al. 2011c^116^ | Data overlaps with other record |
| 117 | Malaty, et al. 2012^117^ | Unable to calculate incidence/prevalence |
| 118 | Malaty, et al. 2013^118^ | Unable to calculate incidence/prevalence |
| 119 | Manguso, et al. 2004^119^ | No rates between 2-17yo |
| 120 | Marin-Jimenez, et al. 2015^120^ | Not English language |
| 121 | Martinez, et al. 2018^121^ | No rates between 2-17yo |
| 122 | Martinez-Osorio, et al. 2017^122^ | No rates between 2-17yo |
| 123 | McErlane, et al. 2008^123^ | EIM not of interest |
| 124 | Mendes, et al. 2007^124^ | No rates between 2-17yo |
| 125 | Mendoza, et al. 2005^125^ | Not English language |
| 126 | Mendoza, et al. 2009^126^ | No rates between 2-17yo |
| 127 | Merrick, et al. 2015a^127^ | Data overlaps with other record |
| 128 | Merrick, et al. 2015b^128^ | Data overlaps with other record |
| 129 | Mocelin, et al. 2015^129^ | No rates between 2-17yo |
| 130 | Moller, et al. 1971^130^ | No rates between 2-17yo |
| 131 | Moravvej, et al. 2008^131^ | No rates between 2-17yo |
| 132 | Morl. 1975^132^ | Not English language |
| 133 | Morlock, et al. 1976^133^ | Not English language |
| 134 | Mosebach, et al. 1995^134^ | Not English language |
| 135 | Nambu, et al. 2016^135^ | Unable to calculate incidence/prevalence |
| 136 | Nuij, et al. 2013^136^ | No rates between 2-17yo |
| 137 | O'Toole, et al. 2012^137^ | No rates between 2-17yo |
| 138 | Okada, et al. 1987^138^ | No rates between 2-17yo |
| 139 | Olbjorn, et al. 2020^139^ | Less than 10 patients |
| 140 | Olsson, et al. 1991^140^ | No rates between 2-17yo |
| 141 | Palm, et al. 2001^141^ | No rates between 2-17yo |
| 142 | Palm, et al. 2002^142^ | No rates between 2-17yo |
| 143 | Papamichael, et al. 2010^143^ | No rates between 2-17yo |
| 144 | Pasternak, et al. 2007^144^ | Less than 10 patients |
| 145 | Patil, et al. 2019^145^ | Unable to calculate incidence/prevalence |
| 146 | Peeters, et al. 2004^146^ | No rates between 2-17yo |
| 147 | Peeters, et al. 2008^147^ | No rates between 2-17yo |
| 148 | Penn, et al. 2013^148^ | No rates between 2-17yo |
| 149 | Platt, et al. 1960^149^ | Unable to calculate incidence/prevalence |
| 150 | Podberesky, et al. 2010^150^ | Unable to calculate incidence/prevalence |
| 151 | Porfito, et al. 2018^151^ | Unable to calculate incidence/prevalence |
| 152 | Powell, et al. 1985^152^ | No rates between 2-17yo |
| 153 | Prathapan, et al. 2019^153^ | Data overlaps with other record |
| 154 | Prathapan, et al. 2020^154^ | Unable to calculate incidence/prevalence |
| 155 | Pusateri, et al. 2013^155^ | Unable to calculate incidence/prevalence |
| 156 | Qari. 2022^156^ | No rates between 2-17yo |
| 157 | Ramzan, et al. 2002^157^ | No rates between 2-17yo |
| 158 | Rasheed, et al. 2015^158^ | Unable to calculate incidence/prevalence |
| 159 | Rasmussen, et al. 1992^159^ | No rates between 2-17yo |
| 160 | Reilly, et al. 2015^160^ | No rates between 2-17yo |
| 161 | Ribaldone, et al. 2015^161^ | No rates between 2-17yo |
| 162 | Ribaldone, et al. 2019^162^ | Not original research article |
| 163 | Rodriguez-Perez, et al. 2008^163^ | Unable to calculate incidence/prevalence |
| 164 | Ronnblom, et al. 2014^164^ | Data overlaps with other record |
| 165 | Roth, et al. 2019^165^ | No rates between 2-17yo |
| 166 | Roth, et al. 2021^166^ | No rates between 2-17yo |
| 167 | Ruemmele, et al. 2016^167^ | Unable to calculate incidence/prevalence |
| 168 | Sahli, et al. 2006^168^ | No rates between 2-17yo |
| 169 | Saigot, et al. 1977^169^ | Not English language |
| 170 | Sams, et al. 1968^170^ | No rates between 2-17yo |
| 171 | Schwartz, et al. 2016^171^ | No rates between 2-17yo |
| 172 | Sdepanian, et al. 2021^172^ | Unable to calculate incidence/prevalence |
| 173 | Shaul, et al. 2021a^173^ | No rates between 2-17yo |
| 174 | Shaul, et al. 2021b^174^ | No rates between 2-17yo |
| 175 | Shentova, et al. 2014^175^ | Data overlaps with other record |
| 176 | Shentova-Eneva, et al. 2019^176^ | Data overlaps with other record |
| 177 | Sheybani, et al. 2014^177^ | Unable to calculate incidence/prevalence |
| 178 | Shitrit, et al. 2015^178^ | No rates between 2-17yo |
| 179 | Shivashankar, et al. 2012^179^ | No rates between 2-17yo |
| 180 | Shrestha, et al. 2021^180^ | Data overlaps with other record |
| 181 | Shrestha, et al. 2022^181^ | Unable to calculate incidence/prevalence |
| 182 | Sladek, et al. 2013^182^ | Data overlaps with other record |
| 183 | Spencer et al. 2023^183^ | Unable to calculate incidence/prevalence |
| 184 | Sridhar. 2018^184^ | EIM not of interest |
| 185 | Subramaniam, et al. 2015^185^ | No rates between 2-17yo |
| 186 | Szakos, et al. 2019^186^ | Unable to calculate incidence/prevalence |
| 187 | Takeuchi, et al. 2015^187^ | Unable to calculate incidence/prevalence |
| 188 | Templier, et al. 2014^188^ | Unable to calculate incidence/prevalence |
| 189 | Templier, et al. 2015a^189^ | Data overlaps with other record |
| 190 | Templier, et al. 2015b^190^ | Unable to calculate incidence/prevalence |
| 191 | Thakkar, et al. 2010^191^ | Unable to calculate incidence/prevalence |
| 192 | Toledo-Maurino, et al. 2020^192^ | No rates between 2-17yo |
| 193 | Topaloglu Demir, et al. 2014^193^ | No rates between 2-17yo |
| 194 | Toy, et al. 2011^194^ | No rates between 2-17yo |
| 195 | Tse, et al. 2018^195^ | No rates between 2-17yo |
| 196 | Turkcapar, et al. 2006^196^ | No rates between 2-17yo |
| 197 | Uchino, et al. 2012^197^ | No rates between 2-17yo |
| 198 | Usoltseva, et al. 2021^198^ | Unable to calculate incidence/prevalence |
| 199 | Vadstrup, et al. 2020^199^ | No rates between 2-17yo |
| 200 | Valentino, et al. 2013^200^ | Data overlaps with other record |
| 201 | Varas, et al. 2016^201^ | Not English language |
| 202 | Vavricka, et al. 2015a^202^ | No rates between 2-17yo |
| 203 | Vavricka, et al. 2015b^203^ | Data overlaps with other record |
| 204 | Vavricka, et al. 2015c^204^ | Data overlaps with other record |
| 205 | Vavricka, et al. 2017^205^ | No rates between 2-17yo |
| 206 | Veloso, et al. 1996^206^ | No rates between 2-17yo |
| 207 | Veres, et al. 2013^207^ | Unable to calculate incidence/prevalence |
| 208 | Vind, et al. 2006^208^ | No rates between 2-17yo |
| 209 | Wang, et al. 2020^209^ | No rates between 2-17yo |
| 210 | Wang, et al. 2007^210^ | No rates between 2-17yo |
| 211 | Wei, et al. 2012^211^ | No rates between 2-17yo |
| 212 | Weng, et al. 2007^212^ | No rates between 2-17yo |
| 213 | Weng, et al. 2022^213^ | No rates between 2-17yo |
| 214 | Wewer, et al. 1991^214^ | No rates between 2-17yo |
| 215 | Wiecek, et al. 2021^215^ | Unable to calculate incidence/prevalence |
| 216 | Yamamoto-Furusho, et al. 2010^216^ |  |
| 217 | Yamamoto Furusho, et al. 2018^217^ | Unable to calculate incidence/prevalence |
| 218 | Yamamoto-Furusho, et al. 2020^218^ | No rates between 2-17yo |
| 219 | Ye, et al. 2011^219^ | No rates between 2-17yo |
| 220 | Yeh, et al. 2021^220^ | Unable to calculate incidence/prevalence |
| 221 | Yeushalmy-Feler, et al. 2018^221^ | Data overlaps with other record |
| 222 | Yilmaz, et al. 2007^222^ | No rates between 2-17yo |
| 223 | Yuksel, et al. 2009^223^ | No rates between 2-17yo |
| 224 | Yuksel, et al. 2011^224^ | No rates between 2-17yo |
| 225 | Zvidi, et al. 2013^225^ | No rates between 2-17yo |

# Supplementary Table 2: Characteristics of included studies.

| **Study ID** | **Study Design** | **Setting/Location/ Study years** | **Number (N) and age range** | **EIMs** | **Outcomes** |
| --- | --- | --- | --- | --- | --- |
| 1. Adamiak 2013^226^ | Prospective cohort | Population-based, Wisconsin, USA 2000-2007 | N=992 (newly diagnosed IBD)  <18 years | Overall EIMs  EN  PG  PSC  AIH | Prevalence at diagnosis |
| 2. Afarideh 2024^227^ | Retrospective cohort | Single-center  Mayo Clinic, Rochester, MN, USA  1999-2017 | N=425  n=276 (CD)  n=149 (UC)  <18 years | EN  PG | Prevalence |
| 3. Aguiar 2017^228^ | Retrospective Cross-sectional | Unknown | N=64 (IBD)  Mean=15.1 years SD=2.8 | Overall EIMs  Sacroiliitis | Prevalence |
| 4. Al Saleem 2015^229^ | Retrospective cohort | Multi-center,  Saudi Arabia  2003-2012 | N=188 (newly diagnosed UC)  <18 years | Overall EIMs  Arthritis | Prevalence at diagnosis |
| 5. Alexopoulou 2012^230^ | Retrospective cross-sectional | Single-center, Athens, Greece  2006-2010 | N=73 (IBD)  n=49 (CD)  n=5 (UC)  n=19 (IC)  7-17 years | Overall EIMs  PSC  AIH | Prevalence |
| 6. Aloi 2013^231^ | Retrospective cohort | Single-center, Rome, Italy 2006-2011 | N=110 (newly diagnosed UC)  0-18 years | Overall EIMs  Axial arthropathies Peripheral arthritis  PSC | Prevalence |
| 7. Alreheili 2018^232^ | Retrospective cohort | Single-center,  Saudi Arabia  2001-2011 | N=66 (IBD)  n=36 (CD)  n=27 (UC)  n=3 (IC)  <14 years | Overall EIMs  Sacroiliitis  Arthritis PSC  EN  PG  Uveitis/Episcleritis  Ankylosing Spondylitis | Prevalence |
| 8. Arcucci 2022^233^ | Retrospective cohort | Multi-center, Argentina  1987-2007,  2007-2017 | N=756 (newly diagnosed IBD)  n=250 (CD)  n=409 (UC)  n=97 (IC)  0-18 years | Overall EIMs  Peripheral arthritis  AIH | Prevalence |
| 9. Ashton 2015^234^ | Retrospective cohort | Single-center, Wessex, UK  2010-2013 | N=172 (newly diagnosed IBD)  n=107 (CD)  n=50 (UC)  n=15 (IC)  <17 years | Overall EIMs  EN  Arthropathy  AIH  PSC | Prevalence at diagnosis |
| 10. Ben Rabeh 2019^235^ | Retrospective cohort | Single-center, Tunisia  2012-2017 | N=14 (IBD)  n=9 (CD)  n=5 (UC)  1.5-14 years | Overall EIMs  PSC  Ankylosing spondylitis  EN | Prevalence |
| 11. Bilgic Dagci 2022^236^ | Retrospective cohort | Single-center, Philadelphia, USA  2015-2018 | N=75 (IBD)  n=54 (CD)  n=6 (UC)  n=15 (IC)  <18 years | Arthritis (peripheral and axial)  Enthesitis | Prevalence |
| 12. Bramuzzo 2016^237^ | Prospective cohort | Population-based, Italy  2009-2014 | N=677 (IBD)  n=292 (CD)  n=360 (UC)  n=25 (IC)  <18 years | Overall EIMs  PSC  AIH  Overlap syndrome | Prevalence |
| 13. Cakir 2015^238^ | Retrospective cohort | Multi-center, Turkey  2009-2015 | N=127 (IBD)  n=29 (CD)  n=90 (UC)  n=8 (IC)  <18 years | Overall EIMs  PSC  Uveitis  EN  AIH | Prevalence |
| 14. Cereser 2022^239^ | Retrospective cross-sectional | Multi-center, Italy  2012-2020 | N=30 (CD)  Mean=14.1 years SD=3.7 | Overall EIMs  Sacroiliitis | Prevalence |
| 15. Chandrakumar 2019^240^ | Prospective cohort | Population-based, Manitoba, Canada  2011-2018 | N=190 (newly diagnosed IBD)  n=95 (CD)  n=90 (UC)  n=5 (IC)  Median=13.71 years  IQR=10.25-15.58) | Overall EIMs  PSC | Incidence Prevalence |
| 16. Cohen 2020^241^ | Retrospective cohort | Single-center, Tel Aviv, Israel  2010-2017 | N=100 (IBD)  n=62 (CD)  n=38 (UC)  <18 years | Overall EIMs  Arthritis  Liver including unknown distribution of AIH, PSC and overlap syndrome | Prevalence |
| 17. Colletti 2019^242^ | Prospective cohort | Multi-center, multi-nation (USA, Canada, Europe)  2007-2018 | N=5501 (IBD)  <18 years | PSC  AIH  Arthritis | Incidence |
| 18. Daniluk 2021^243^ | Retrospective cohort | Single-center, Poland  2013-2018 | N=119 (newly diagnosed IBD)  n=42 (CD)  n=77 (UC)  1-17 years | Overall EIMs  PSC  ASC  AIH | Prevalence |
| 19. Dass 2023^244^ | Retrospective cohort | Single-center, Wayne County, Michigan, USA  2013-2023 | N=127 (IBD)  n=75 (CD)  n=50 (UC)  n=2 (IC)  <18 years | Overall EIMs  Ankylosing spondylitis  Arthritis  EN  PSC  AIH | Prevalence |
| 20. Deneau 2013^245^ | Retrospective cohort | Population-based, Utah, USA  1986-2011 | N=607 (IBD)  n=317 (CD)  n=262 (UC)  n=28 (IC)  0-18 years | PSC  AIH  ASC | Prevalence |
| 21. Derfalvi 2022^246^ | Prospective cross-sectional | Single-center, Budapest, Hungary | N=82 (CD)  0-18 years | Sacroiliitis  Enthesitis  Arthritis | Prevalence |
| 22. Dimakou 2015^247^ | Retrospective cohort | Single-center, Athens, Greece  1981-2011 | N=483 (IBD)  n=167 (CD)  n=267 (UC)  n=49 (IC)  0.5-18 years | Arthritis  PSC  AIH  Overlap syndrome | Prevalence |
| 23. Dong 2023^248^ | Retrospective cohort | Multi-center, Saxony, Germany 2000-2014 | N=338 (newly diagnosed CD)  0-14 years | Overall EIMs  PSC | Prevalence |
| 24. Dotson 2010^249^ | Prospective Cohort | Multi-center, USA and Canada  2002-2007 | N=1009 (IBD)  n=728 (CD)  n=281 (UC)  <16 years | Overall EIMs  Ankylosing spondylitis  Arthritis  EN  PG  PSC | Prevalence |
| 25. Dzongowski 2023^250^ | Prospective cohort | Multi-center  Canada  2014-2021 | N=1330 (IBD)  n=824 (CD)  n=382 (UC)  n=124 (IC)  2-17 years | Overall EIMs  Enthesitis  Arthritis  Sacroiliitis Ankylosing spondylitis | Prevalence |
| 26. Fallahi 2009^251^ | Retrospective cohort | Single-center, Tehran, Iran | N=59 (IBD)  n=19 (CD)  n=23 (UC)  n=17 (IC)  <18 years | EN  PSC  AIH  Arthritis | Prevalence |
| 27. Gerenli 2021^252^ | Prospective cross-sectional | Single-center, Istanbul, Turkey | N=31 (IBD)  n=11 (CD)  n=17 (UC)  n=3 (IC)  7-18 years | Overall EIMs  Enthesitis | Prevalence |
| 28. Gerenli 2022^253^ | Prospective cross-sectional | Single-center, Istanbul, Turkey | N=27 (IBD)  n=11 (CD)  n=14 (UC)  n=2 (IC)  7-18 years | Overall EIMs  Sacroiliitis  EN | Prevalence |
| 29. Ghersin 2020^254^ | Retrospective cohort | Population-based, Israel  2004-2016 | N=2372 (IBD)  n=1612 (CD)  n=760 (UC)  Median=17.1 years  IQR= 16.7–17.3 years | Arthritis  Uveitis  PSC  AIH | Prevalence |
| 30. Giani 2020^255^ | Retrospective cohort | Single-center, Florence, Italy  2010-2018 | N=34 (IBD)  n=32 (CD)  n=2 (UC)  Mean=14.3 years | Overall EIMs  Sacroiliitis | Prevalence |
| 31. Goyal 2014^256^ | Retrospective cohort | Multi-center, USA and Canada  2002-2013 | N=1569 (newly diagnosed IBD)  n=1075 (CD)  n=401 (UC)  n=93 (IC)  <16 years | Overall EIMs  PSC  AIH  Overlap syndrome | Prevalence |
| 32. Greuter 2017^257^ | Retrospective cohort | Population-based, Switzerland  2008 | N=329 (IBD)  n=173 (CD)  n=156 (UC/IBD-U)  <18 years | Overall EIMs  Arthropathy  Arthritis  Uveitis  EN  PG  PSC | Prevalence |
| 33. Guariso 2010^258^ | Retrospective/ Prospective cohorts | Single-center, Padua, Italy  1994-2008 | N=133 (IBD)  n=67 (CD)  n=58 (UC)  n=8 (IC)  0-17 years | EN | Prevalence |
| 34. Hlouskova 2019^259^ | Retrospective cohort | Single-center, Brno, Czech Republic  2002-2018 | N=412 (IBD)  8-17 years | ASC  PSC  Overlap syndrome | Prevalence |
| 35. Hofley 1993^260^ | Prospective cross-sectional | Single-center, Toronto, Canada  1992 | N=147 (IBD)  n=97 (CD)  n=50 (UC)  4-17 years | Overall EIMs  Uveitis | Prevalence |
| 36. Horton 2012^261^ | Prospective cross-sectional | Single-center, Philadelphia, USA  2011 | N=43 (IBD)  n=32 (CD)  n=1 (UC)  n=10 (IC)  4-21 years | Overall EIMs  Enthesitis | Prevalence |
| 37.Isa 2018^262^ | Retrospective cohort | Single-center, Bahrain  1984-2017 | N=51 (CD)  <18 years | Arthritis  EN | Prevalence |
| 38. Ivkovic 2020^263^ | Prospective cohort | Single-center, Croatia  2016-2017 | N=51 (IBD)  n=19 (CD)  n=28 (UC)  n=4 (IC)  0-18 years | Overall EIMs  Arthritis  EN  PSC  AIH | Prevalence |
| 39. Jang 2021^264^ | Retrospective cohort | Multi-center, Korea  2010-2017 | N=172 (IBD)  n=137 (CD)  n=35 (UC)  < 18 years | Overall EIMs  Ankylosing spondylitis  EN  PSC  PG  Uveitis | Prevalence |
| 40. Jang 2022^265^ | Retrospective cohort | Single-center, Korea  1987-2013 | N=208 (UC)  <18 years | Arthritis  EN  PG  PSC | Prevalence |
| 41. Jashmi 2022^266^ | Retrospective cohort | Population-based, UK  2011-2022 | N=770 (IBD) | Overall EIMs  Arthritis | Prevalence |
| 42. Jose 2009^267^ | Retrospective cohort | Multi-center, USA  2000-2003 | N=1649 (IBD)  n=1007 (CD)  n=471 (UC)  n=171 (IC)  <18 years | Overall EIMs  Arthritis  Uveitis  EN  PG  PSC  AIH | Prevalence |
| 43. Kanavaki 2023^268^ | Retrospective cohort | Single-center, Athens Greece  2018-2023 | N=70 (IBD)  n=48 (CD)  n=19 (UC)  n=3 (IC)  5-17 years | Overall EIMs  PSC  AIH  Overlap syndrome | Prevalence |
| 44. Kim 2017^269^ | Retrospective cohort | Single-center, Seoul, Korea  1988-2013 | N=220 (UC)  <18years | Overall EIMs  PSC | Prevalence |
| 45. Klemenak 2024^270^ | Retrospective cohort | Single-center, North-East Slovenia  2014-2023 | N=87 (IBD)  n=38 (CD)  n=42 (UC)  n=7 (IC)  4-18 years | Overall EIMs  Arthritis  Uveitis  PSC | Prevalence |
| 46. Kourti 2023^271^ | Retrospective cohort | Single-center, Athens Greece  2018-2023 | N=70 (IBD)  n=48 (CD)  n=19 (UC)  n=3 (IC)  5-17 years | Overall EIMs | Prevalence |
| 47. Kwon 2022^272^ | Retrospective cohort | Single-center, Korea  2003-2021 | N=142 (UC)  <18 years | Overall EIMs  Arthropathy  Sacroiliitis  Arthritis  Uveitis  PG  PSC | Prevalence |
| 48. Lagercrantz 1958^273^ | Retrospective cohort | Single-center, Stockholm, Sweden  1951-1956 | N=48 (UC)  1-15 years | EN | Prevalence |
| 49. Lee 2016^274^ | Retrospective cohort | Single-center, Busan, Korea  1995-2011 | N=73 (CD)  <20 years | Arthritis  Uveitis  EN | Prevalence |
| 50. Lekovic 2011^275^ | Retrospective cohort | Single-center, Serbia  2000-2007 | N=17 (UC)  3-17 years | PG  AIH | Prevalence |
| 51. Levy 2019^276^ | Retrospective cohort | Single-center, Israel  1985-2016 | N=715 (IBD)  6-18 years | Overall EIMs  Sacroiliitis  Arthritis | Prevalence |
| 52. Lim 2013^277^ | Retrospective cohort | Single-center, Western Australia  2004-2013 | N=157 (IBD) | AIH  PSC  ASC | Prevalence |
| 53. Lindsley 1974^278^ | Retrospective cohort | Single-center, Seattle, USA  1960-1970 | N=136 (IBD)  n=86 (CD)  n=50 (UC)  <19 years | Ankylosing spondylitis  Arthritis | Prevalence |
| 54. Malmborg 2016^279^ | Retrospective cohort | Population-based, Northern Stockholm County, Sweden  1990-2011 | N=280 (IBD)  <16 years | Overall EIMs  PSC  AIH  ASC | Prevalence |
| 55. Maniscalco 2024^280^ | Retrospective cohort | Multi-center, Italy  2009-2022 | N=4229 (IBD)  n=1843 (CD)  n=2109 (UC)  n=277 (IC)  0-18 years | Uveitis | Prevalence |
| 56. Mataly 2013^281^ | Retrospective cohort | Single-center, Houston, Texas, USA  1986-2003 | N=115 (UC) | Arthritis  PSC | Prevalence |
| 57. Matar 2017^282^ | Retrospective cohort | Single-center, Israel  2002-2015 | N=184 (IBD)  n=129 (CD)  n=46 (UC)  n=9 (IC)  0-18 years | Arthritis  PSC | Prevalence |
| 58. Medynska-Przeczek 2023^283^ | Retrospective cohort | Kraków, Poland | N=21 (newly diagnosed CD)  3-17 years | PSC  AIH | Prevalence |
| 59. Merrick 2017^284^ | Prospective cohort | Multi-center, Scotland, UK  2012-2015 | N=809 (IBD)  <17 years | Ankylosing spondylitis  PSC  Arthritis | Prevalence |
| 60. Naviglio 2017^285^ | Retrospective cross-sectional | Single-center, Trieste, Italy  2014-2016 | N=94 (IBD)  n=46 (CD)  n=46 (UC)  n=2 (IC)  0-17 years | Uveitis | Prevalence |
| 61. Nemeth 1990^286^ | Retrospective cohort | Single-center, Sweden  1973-1985 | N=46 (IBD)  n=12 (CD)  n=34 (UC)  0-17 years | PSC | Prevalence |
| 62. Niewiem 2019^287^ | Retrospective cohort | Single-center, Poland  2005-2017 | N=287 (IBD)  n=140 (CD)  n=147 (UC)  2-18 years | Overall EIMs  Arthropathy  Arthritis  EN  PSC  Overlap syndrome | Prevalence |
| 63. Nir 2017^288^ | Retrospective cohort | Single-center, Israel  2000-2016 | N=430 (IBD)  n=301 (CD)  n=129 (UC/IC)  0-18 years | Arthritis | Prevalence |
| 64. Noble-Jamieson 2013^289^ | Retrospective cohort | Single-center, Cambridge, UK  2004-2012 | N=52 (UC/IC)  9-16 years | Overall EIMs  PSC Overlap syndrome | Prevalence |
| 65. Ong 1994^290^ | Retrospective cohort | Single-center, Sydney, Australia  1973-1991 | N=130 (IBD)  n=47 (CD)  n=83 (UC) | PSC | Prevalence |
| 66. Ong 2014^291^ | Retrospective cohort | Single-center, Singapore  1996-2013 | N=123 (IBD)  n=82 (CD)  n=28 (UC)  n=13 (IC)  1.5-17 years | PSC | Prevalence |
| 67. Ouldali 2018^292^ | Retrospective cohort | Single-center, Paris, France  2005-2016 | N=272 (CD)  <18 years | Overall EIMs  Arthritis | Prevalence |
| 68. Passo 1986^293^ | Retrospective cohort | Single-center, Indianapolis, USA  10 yrs | N=102 (IBD)  n=58 (CD)  n=44 (UC)  2-16 years | Overall EIMs  Arthritis | Prevalence |
| 69. Pytrus 2011^294^ | Retrospective cohort | Single-center, Wroclaw, Poland  2005-2010 | N=143 (IBD)  n=86 (CD)  n=57 (UC)  3-18 years | EN  PG | Prevalence |
| 70. Rahmani 2022^295^ | Retrospective cross-sectional | Single-center, Tehran, Iran  2019 | N=73 (IBD)  n=33 (CD)  n=40 (UC)  2-18 years | Arthritis  Ankylosing spondylitis  Uveitis  EN  PG | Prevalence |
| 71. Rajwal 2004^296^ | Retrospective cohort | Single-center, Leeds, UK  1996-2001 | N=30 (UC)  2-15 years | Arthritis  PSC | Prevalence |
| 72. Rohani 2021^297^ | Retrospective cross-sectional | Single-center, Tehran, Iran  2015-2019 | N=120 (IBD) | PSC  AIH  ASC | Prevalence |
| 73. Ronnblom 2015^298^ | Retrospective cohort | Population-based, Sweden  2005-2009 | N=92 (IBD) | PSC  AIH | Prevalence |
| 74. Ruiz 2009^299^ | Retrospective cohort | Single-center, Buenos Aires, Argentina  2006-2007 | N=424 (IBD)  n=103 (CD)  n=263 (UC)  n=58 (IC)  0-21 years | Overall EIMs  Arthritis | Prevalence |
| 75. Rychwalski 1997^300^ | Prospective cross-sectional | St. Louis, USA  1994-1995 | N=32 (IBD)  n=18 (CD)  n=14 (UC)  1-19 years | Uveitis | Prevalence |
| 76. Sassine 2022^301^ | Retrospective cohort | Single-center,  Quebec, Canada  2009-2019 | N=654 (CD)  <18 years | Arthritis  Uveitis  EN  PSC  AIH | Prevalence |
| 77. Schaefer 2018^302^ | Prospective cohort | Multi-center, USA  2006-2016 | N=17587 (IBD)  Mean=14 years at uveitis diagnosis | Uveitis | Prevalence |
| 78. Schoepfer 2022^303^ | Prospective cohort | Population-based, Switzerland  2006-2019 | N=184 (UC)  2-18 years | Overall EIMs  Ankylosing spondylitis  Arthritis  Sacroiliitis  Uveitis/iritis  EN  PG  PSC | Prevalence |
| 79. Seo 1992^304^ | Retrospective cohort | Single-center,  Seoul, Korea  1987-1991 | N=22 (IBD)  n=12 (CD)  n=10 (UC)  2-14 years | Arthritis  EN  PG | Prevalence |
| 80. Shentova-Eneva 2019^305^ | Retrospective cohort | Single-center,  Sofia, Bulgaria  2011-2018 | N=91 (IBD)  n=40 (CD)  n=51 (UC)  2-17 years | Overall EIMs  Arthritis  PG | Prevalence |
| 81. Sladek 2014^306^ | Prospective cohort | Krakow, Poland | N=585 (newly diagnosed IBD)  n=368 (CD)  n=140 (UC)  n=76 (IC) | PSC | Prevalence |
| 82. Sonavane 2018^307^ | Retrospective cohort | Single-center, Mumbai, India  2004-2016 | N=65 (IBD)  n=24 (CD)  n=41 (UC)  4-19 years | Sacroiliitis  PG | Prevalence |
| 83. Stenhammar 1994^308^ | Retrospective cohort | Single-center, Sweden  1990-1994 | N=31 (IBD)  n=9 (CD)  n=14 (UC)  n=8 (IC) | PSC | Prevalence |
| 84. Taskin 2023^309^ | Retrospective cohort | Single-center,  Turkey  2017-2021 | N=57 (IBD)  n=25 (CD)  n=32 (UC) | Overall EIMs  Arthritis  EN | Prevalence |
| 85. Valentino 2015^310^ | Retrospective cohort | Single-center, Toronto, Canada  2000-2011 | N=300 (IBD)  n=163 (CD)  n=100 (UC)  n=37 (IC)  <18 years | PSC  ASC | Prevalence |
| 86. Van Der Feen 2014^311^ | Prospective cohort | Single-center, UK  2002-2013 | N=504 (newly diagnosed IBD)  n=313 (CD)  n=137 (UC)  n=54 (IC) | ASC  AIH | Prevalence |
| 87. Yousif 2023a^312^  Yousif 2023b^313^ | Retrospective cohort | Multi-center, USA | N=32497 (IBD)  <21 years | EN  PG  Arthritis  Uveitis | Prevalence |
| 88. Yu 2023^314^ | Retrospective cohort | Single-center, Philadelphia, USA  2009-2021 | N=315 (IBD)  n=209 (CD)  n=51 (UC)  n=55 (IC)  <18 years | Uveitis | Prevalence |
| 89. Zhou 2016^315^ | Retrospective cohort | Single-center, Shanghai, China  2001-2012 | N=49 (IBD)  n=41 (CD)  n=8 (UC)  <18 years | Arthritis | Prevalence |
| 90. Zong 2019^316^ | Retrospective cohort | Single-center, Philadelphia, USA  2013-2018 | N=70 (IBD)  n=40 (CD)  n=16 (UC)  n=14 (IC)  Mean=9.8 years | Uveitis  EN | Prevalence |

Abbreviations: AIH, autoimmune hepatitis; ASC, autoimmune sclerosing cholangitis; CD, Crohn’s disease; EIM, extra-intestinal manifestation; EN, erythema nodosum; IBD, inflammatory bowel disease; IBD-U, inflammatory bowel disease unclassified; IC, indeterminate colitis; IQR, interquartile range; PG, pyoderma gangrenosum; PSC, primary sclerosing cholangitis; SD, standard deviation; UC, ulcerative colitis.

# Supplementary Table 3: Quality assessment of cohort and cross-sectional studies (Newcastle-Ottawa scale)

| **Study** | **Selection** | **Comparability** | **Outcomes** | **Total** |
| --- | --- | --- | --- | --- |
| 1. Adamiak 2013 | 3 | 0 | 2 | 5 |
| 2. Afarideh 2024 | 3 | 0 | 2 | 5 |
| 3. Aguiar 2017 | 2 | 0 | 2 | 4 |
| 4. Al Saleem 2015 | 3 | 0 | 2 | 5 |
| 5. Alexopoulou 2012 | 3 | 0 | 2 | 5 |
| 6. Aloi 2013 | 3 | 1 | 2 | 6 |
| 7. Alreheili 2018 | 3 | 0 | 2 | 5 |
| 8. Arcucci 2022 | 3 | 0 | 3 | 6 |
| 9. Ashton 2015 | 3 | 0 | 2 | 5 |
| 10. Ben Rabeh 2019 | 2 | 0 | 2 | 4 |
| 11. Bilgic Dagci 2022 | 3 | 2 | 3 | 8 |
| 12. Bramuzzo 2016 | 3 | 2 | 2 | 7 |
| 13. Cakir 2015 | 3 | 0 | 2 | 5 |
| 14. Cereser 2022 | 3 | 0 | 3 | 6 |
| 15. Chandrakumar 2019 | 3 | 2 | 2 | 7 |
| 16. Cohen 2020 | 3 | 2 | 2 | 7 |
| 17. Colletti 2019 | 3 | 0 | 2 | 5 |
| 18. Daniluk 2021 | 3 | 0 | 2 | 5 |
| 19. Dass 2023 | 3 | 0 | 2 | 5 |
| 20. Deneau 2013 | 3 | 0 | 2 | 5 |
| 21. Derfalvi 2022 | 3 | 1 | 3 | 7 |
| 22. Dimakou 2015 | 3 | 0 | 1 | 4 |
| 23. Dong 2023 | 3 | 2 | 3 | 8 |
| 24. Dotson 2010 | 3 | 0 | 2 | 5 |
| 25. Dzongowski 2023 | 3 | 2 | 2 | 7 |
| 26. Fallahi 2009 | 3 | 0 | 2 | 5 |
| 27. Gerenli 2021 | 3 | 0 | 3 | 6 |
| 28. Gerenli 2022 | 3 | 0 | 3 | 6 |
| 29. Ghersin 2020 | 3 | 2 | 2 | 7 |
| 30. Giani 2020 | 3 | 0 | 1 | 4 |
| 31. Goyal 2014 | 3 | 0 | 2 | 5 |
| 32. Greuter 2017 | 3 | 2 | 2 | 7 |
| 33. Guariso 2010 | 3 | 2 | 2 | 7 |
| 34. Hlouskova 2019 | 3 | 0 | 1 | 4 |
| 35. Hofley 1993 | 3 | 0 | 1 | 4 |
| 36. Horton 2012 | 3 | 0 | 3 | 6 |
| 37.Isa 2018 | 3 | 0 | 2 | 5 |
| 38. Ivkovic 2020 | 3 | 0 | 1 | 4 |
| 39. Jang 2021 | 3 | 2 | 2 | 7 |
| 40. Jang 2022 | 3 | 0 | 2 | 5 |
| 41. Jashmi 2022 | 2 | 0 | 1 | 3 |
| 42. Jose 2009 | 3 | 2 | 2 | 7 |
| 43. Kanavaki 2023 | 2 | 0 | 1 | 3 |
| 44. Kim 2017 | 3 | 0 | 2 | 5 |
| 45. Klemenak 2024 | 3 | 0 | 1 | 4 |
| 46. Kourti 2023 | 2 | 0 | 1 | 3 |
| 47. Kwon 2022 | 3 | 0 | 2 | 5 |
| 48. Lagercrantz 1958 | 2 | 0 | 0 | 2 |
| 49. Lee 2016 | 3 | 0 | 2 | 5 |
| 50. Lekovic 2011 | 2 | 0 | 0 | 2 |
| 51. Levy 2019 | 3 | 0 | 1 | 4 |
| 52. Lim 2013 | 3 | 0 | 1 | 4 |
| 53. Lindsley 1974 | 3 | 0 | 2 | 5 |
| 54. Malmborg 2016 | 3 | 0 | 2 | 5 |
| 55. Maniscalco 2024 | 3 | 0 | 3 | 6 |
| 56. Mataly 2013 | 3 | 2 | 3 | 8 |
| 57. Matar 2017 | 3 | 2 | 2 | 7 |
| 58. Medynska-Przeczek 2023 | 2 | 0 | 0 | 2 |
| 59. Merrick 2017 | 3 | 0 | 2 | 5 |
| 60. Naviglio 2017 | 3 | 0 | 2 | 5 |
| 61. Nemeth 1990 | 2 | 0 | 2 | 4 |
| 62. Niewiem 2019 | 3 | 0 | 2 | 5 |
| 63. Nir 2017 | 3 | 2 | 2 | 7 |
| 64. Noble-Jamieson 2013 | 3 | 0 | 2 | 5 |
| 65. Ong 2014 | 3 | 0 | 1 | 4 |
| 66. Ong 1994 | 2 | 0 | 1 | 3 |
| 67. Ouldali 2018 | 3 | 2 | 3 | 8 |
| 68. Passo 1986 | 3 | 0 | 2 | 5 |
| 69. Pytrus 2011 | 2 | 0 | 0 | 2 |
| 70. Rahmani 2022 | 3 | 0 | 3 | 6 |
| 71. Rajwal 2004 | 3 | 0 | 3 | 6 |
| 72. Rohani 2021 | 3 | 0 | 2 | 5 |
| 73. Ronnblom 2015 | 3 | 0 | 1 | 4 |
| 74. Ruiz 2009 | 2 | 0 | 1 | 3 |
| 75. Rychwalski 1997 | 3 | 0 | 0 | 3 |
| 76. Sassine 2022 | 3 | 1 | 1 | 5 |
| 77. Schaefer 2018 | 3 | 0 | 2 | 5 |
| 78. Schoepfer 2022 | 3 | 2 | 2 | 7 |
| 79. Seo 1992 | 2 | 0 | 2 | 4 |
| 80. Shentova-Eneva 2019 | 2 | 0 | 1 | 3 |
| 81. Sladek 2014 | 2 | 0 | 0 | 2 |
| 82. Sonavane 2018 | 3 | 0 | 2 | 5 |
| 83. Stenhammar 1994 | 2 | 0 | 0 | 2 |
| 84. Taskin 2023 | 3 | 0 | 1 | 4 |
| 85. Valentino 2015 | 3 | 2 | 2 | 7 |
| 86. Van Der Feen 2014 | 3 | 0 | 2 | 5 |
| 87. Yousif 2023a  Yousif 2023b | 3 | 2 | 1 | 6 |
| 88. Yu 2023 | 2 | 0 | 0 | 2 |
| 89. Zhou 2016 | 3 | 0 | 1 | 4 |
| 90. Zong 2019 | 3 | 0 | 1 | 4 |

Newcastle Ottawa scores ≥ 7 correspond with high quality and a lower risk of bias. Scores < 7 correspond with low quality and a higher risk of bias.

# References

1 Aadland E, Schrumpf E, Fausa O, et al. Primary sclerosing cholangitis: a long-term follow-up study. *Scandinavian Journal of Gastroenterology*. 1987;22:655-664.

2 Abbasian J, Martin TM, Patel S, et al. Immunologic and genetic markers in patients with idiopathic ocular inflammation and a family history of inflammatory bowel disease. *American Journal of Ophthalmology*. Research Support, N.I.H., Extramural

Research Support, Non-U.S. Gov't. 2012;154:72-77.

3 Adeniyi OF, Sr., Odeghe E, Olatona FA, et al. Inflammatory Bowel Disease in Children: Experience and Constraints in a Resource-limited Setting. *Cureus*. 2020;12:e7848.

4 Aguilera M, Vega V, Soliman A, et al. A Retrospective Study of the National Inpatient Sample (Nis) Demonstrates Divergence in Characteristics and Outcomes of Pediatric Inflammatory Bowel Disease (Ibd) in Hispanic Patients Vs Non-Hispanic Whites. *Journal of Pediatric Gastroenterology and Nutrition*. 2023;77(1 Supplement 1):S296-S297.

5 Al-Mendalawi MD. Extraintestinal manifestations of pediatric inflammatory bowel disease in Saudi Arabia. *Saudi Journal of Gastroenterology*. 2018;24(5):307.

6 Al-Mendalawi MD. Pediatric Crohn's disease in Bahrain. *Oman Medical Journal*. 2019;34(3):268.

7 Aletaha D, Epstein AJ, Skup M, et al. Risk of Developing Additional Immune-Mediated Manifestations: A Retrospective Matched Cohort Study. *Advances in Therapy*. Research Support, Non-U.S. Gov't. 2019;36:1672-1683.

8 Alkhouri N, Hupertz V, Mahajan L. Adalimumab treatment for peristomal pyoderma gangrenosum associated with Crohn's disease. *Inflammatory Bowel Diseases*. Case Reports

Letter. 2009;15:803-806.

9 Almaas R. Diagnosing autoimmune hepatitis in paediatric patients - we still haven't found what we are looking for. *Acta Paediatrica, International Journal of Paediatrics*. 2019;108(9):1562-1563.

10 Alreheili K, Almehaidib A, Banemi M, et al. Clinical presentation of inflammatory bowel disease in Saudi children (Single centre experience). *International Journal of Pediatrics and Adolescent Medicine*. 2016;3(4):175-179.

11 Amouri M, Masmoudi A, Ammar M, et al. Sweet's syndrome: a retrospective study of 90 cases from a tertiary care center. *International Journal of Dermatology*. Case Reports. 2016;55:1033-1039.

12 Araujo D, Neves IM, Antunes H. Primary Sclerosing Cholangitis-importance of its diagnosis in children. *Journal of Pediatric Gastroenterology and Nutrition*. 2022;74(2 Supplement 2):732.

13 Assa A, Nir O, Rinawi F, et al. Phenotypic features and long-term outcomes of pediatric inflammatory bowel disease patients with arthritis and arthralgia. *Journal of Pediatric Gastroenterology and Nutrition*. 2017;65(Supplement 1):S13.

14 Assa A, Gershin I, Daher S, et al. Comorbidities in adolescents with inflammatory bowel disease: Findings from a populationbased cohort. *Journal of Pediatric Gastroenterology and Nutrition*. 2018;66(Supplement 2):48.

15 Aziz DA, Moin M, Majeed A, et al. Paediatric Inflammatory Bowel Disease: Clinical Presentation and Disease Location. *Pakistan Journal of Medical Sciences*. 2017;33:793-797.

16 Bandinelli F, Terenzi R, Giovannini L, et al. Occult radiological sacroiliac abnormalities in patients with inflammatory bowel disease who do not present signs or symptoms of axial spondylitis. *Clinical & Experimental Rheumatology*. 2014;32:949-952.

17 Barreiro-De Acosta M, Enrique Dominguez-Munoz J, Concepcion Nunez-Pardo De Vera M, et al. Relationship between clinical features of Crohn's disease and the risk of developing extraintestinal manifestations. *European Journal of Gastroenterology and Hepatology*. 2007;19(1):73-78.

18 Barrie A, Mourabet ME, Weyant K, et al. Recurrent blood eosinophilia in ulcerative colitis is associated with severe disease and primary sclerosing cholangitis. *Digestive Diseases & Sciences*. Research Support, N.I.H., Extramural

Research Support, Non-U.S. Gov't. 2013;58:222-228.

19 Batticciotto A, Dilillo D, Antivalle M, et al. Prevalence of subclinical entheseal involvement in patients with paediatric inflammatory bowel disease: An ultrasonographic study. *Arthritis and Rheumatology*. 2014;10):S60-S61.

20 Batticciotto A, Dilillo D, Antivalle M, et al. Ultrasonographic evaluation of subclinicial enthesitis in patients affected by pediatric inflammatory bowel disease. *Annals of the Rheumatic Diseases*. 2015;74(SUPPL 2):877.

21 Beauvais C, Le Quintrec JL, Prier A, et al. [Destructive arthritis of the hip in Crohn disease. 10 cases]. *Presse Medicale*. 1995;24:1555-1558.

22 Ben Abdesslem N, Mahjoub A, Sayadi S, et al. Ocular manifestations of crohn's disease. *Tunisie Medicale*. 2019;97:692-697.

23 Bennett RA, Rubin PH, Present DH. Frequency of inflammatory bowel disease in offspring of couples both presenting with inflammatory bowel disease. *Gastroenterology*. 1991;100:1638-1643.

24 Biedermann L, Renz L, Fournier N, et al. Uveitis manifestations in patients of the Swiss Inflammatory Bowel Disease Cohort Study. *Therap Adv Gastroenterol*. 2019;12:1756284819865142.

25 Boberg KM, Schrumpf E, Fausa O, et al. Hepatobiliary disease in ulcerative colitis. *Scandinavian Journal of Gastroenterology*. 1994;29(8):744-752.

26 Bornman PC, Lemmer ER, Robson SC, et al. Asymptomatic primary sclerosing cholangitis in association with inflammatory bowel disease. *Journal of Clinical Gastroenterology*. Letter. 1993;16:263-264.

27 Boskovic A, Kitic I, Prokic D, et al. Epidemiology of pediatric chronic inflammatory bowel disease in Serbia, 2005 2009. *Journal of Crohn's and Colitis*. 2011;5(1):S149.

28 Bracci F, Girolami E, Torre G, et al. Pediatric ulcerative colitis associated with primary sclerosing cholangitis: a distinct form of inflammatory bowel disease? The clinical relevance of anti-lactoferrin antibodies. *Inflammatory Bowel Diseases*. Letter

Comment. 2013;19:E56-57.

29 Brakenhoff LK, Stomp W, van Gaalen FA, et al. Magnetic resonance imaging of the hand joints in patients with inflammatory bowel disease and arthralgia: a pilot study. *Scandinavian Journal of Rheumatology*. Research Support, Non-U.S. Gov't. 2014;43:416-418.

30 Bramuzzo M, Martelossi S, Barabino A, et al. Inflammatory bowel disease characteristics in children with associated immunomediated hepatobiliary diseases: A comparative study from the Italian paediatric ibd registry. *Digestive and Liver Disease*. 2014;46(SUPPL 3):e90.

31 Bramuzzo M, Lionetti P, Miele E, et al. Phenotype and natural history of children with coexistent inflammatory bowel disease and coeliac disease. *Journal of Pediatric Gastroenterology and Nutrition*. 2021;72(SUPPL 1):85-86.

32 Broome U, Glaumann H, Hellers G, et al. Liver disease in ulcerative colitis: an epidemiological and follow up study in the county of Stockholm. *Gut*. Research Support, Non-U.S. Gov't. 1994;35:84-89.

33 Bruining DH, Siddiki HA, Fletcher JG, et al. Prevalence of penetrating disease and extraintestinal manifestations of Crohn's disease detected with CT enterography. *Inflammatory Bowel Diseases*. Research Support, Non-U.S. Gov't. 2008;14:1701-1706.

34 Burgos K, Blanco A, Emanuelli N, et al. A comparison of pediatric vs adult onset IBD in Puerto Rican Hispanics. *American Journal of Gastroenterology*. 2019;114(Supplement 1):S3.

35 Card TR, Langan SM, Chu TP. Extra-Gastrointestinal Manifestations of Inflammatory Bowel Disease May Be Less Common Than Previously Reported. *Digestive Diseases & Sciences*. Research Support, Non-U.S. Gov't. 2016;61:2619-2626.

36 Cardile S, Candusso M, Papadatou B, et al. Coexistence of inflammatory bowel disease (IBD) and primary sclerosing cholangitis (PSC) in children: A clinical and pathogenetic intrigued model. *Digestive and Liver Disease*. 2015;47(SUPPL 4):e255-e256.

37 Cardile S, Candusso M, Alterio T, et al. Inflammatory bowel diseases associated with primary sclerosing cholangitis in children: A ten years case-control retrospective study. *Journal of Pediatric Gastroenterology and Nutrition*. 2019;68(Supplement 1):502.

38 Cardile S, Chiaro A, Comito D, et al. Arthropathies associated to inflammatory bowel disease (IBD): A retrospective survey. *Journal of Crohn's and Colitis*. 2012;6(SUPPL 1):S56.

39 Caspersen S, Elkjaer M, Riis L, et al. Infliximab for Inflammatory Bowel Disease in Denmark 1999-2005: Clinical Outcome and Follow-Up Evaluation of Malignancy and Mortality. *Clinical Gastroenterology and Hepatology*. 2008;6(11):1212-1217.

40 Catassi G, D'Arcangelo G, Norsa L, et al. Long-Term Outcome of Very Early Onset Inflammatory Bowel Disease Associated with Primary Sclerosing Cholangitis: A Multicenter Study from the Pediatric Ibd Porto Group of Espghan. *Journal of Pediatric Gastroenterology and Nutrition*. 2023;76(Supplement 1):79-80.

41 Catassi G, D'Arcangelo G, Norsa L, et al. Outcome of Very Early Onset Inflammatory Bowel Disease Associated With Primary Sclerosing Cholangitis: A Multicenter Study From the Pediatric IBD Porto Group of ESPGHAN. *Inflammatory Bowel Diseases*. 2023;28:28.

42 Chan J, Sari I, Salonen D, et al. Prevalence of Sacroiliitis in Inflammatory Bowel Disease Using a Standardized Computed Tomography Scoring System. *Arthritis care & research*. Research Support, Non-U.S. Gov't. 2018;70:807-810.

43 Chapuy L, Godin D, Patey N, et al. Non-Monogenic Very Early Onset Inflammatory Bowel Disease: A Tertiary Center Six Years Retrospective Experience. Gastroenterology 2020;158:S970-S970. *Gastroenterology*. 2020;158:S970.

44 Cheikhna F, Omari H, Tahiri M, et al. Hepatobiliary manifestations associated to chronic inflammatory bowel disease. *Turkish Journal of Gastroenterology*. 2019;30(Supplement 3):S514.

45 Chew KS, Aw MM, Huang JG, et al. Very early onset inflammatory bowel disease in Asian children. *Journal of Pediatric Gastroenterology and Nutrition*. 2019;69(Supplement 1):56.

46 Chlebowczyk U, Wiecek S, Kajor M, et al. Primary sclerosing cholangitis in the course of inflammatory bowel disease in children. *Journal of Crohn's and Colitis*. 2012;6(SUPPL 1):S44.

47 Conti F, Borrelli O, Anania C, et al. Chronic intestinal inflammation and seronegative spondyloarthropathy in children. *Digestive & Liver Disease*. Comparative Study. 2005;37:761-767.

48 Cordes F, Laumeyer T, Gers J, et al. Distinct Disease Phenotype of Ulcerative Colitis in Patients With Coincident Primary Sclerosing Cholangitis: Evidence From a Large Retrospective Study With Matched Cohorts. *Diseases of the Colon & Rectum*. Comparative Study. 2019;62:1494-1504.

49 Coutzac C, Chapuis J, Poullenot F, et al. Association Between Infliximab Trough Levels and the Occurrence of Paradoxical Manifestations in Patients with Inflammatory Bowel Disease: a Case-Control Study. *Journal of Crohn's & colitis*. Clinical Trial. 2015;9:982-987.

50 Csizmadia I, Cooper S, Ekpotu LP, et al. Risk factors for developing paediatric inflammatory bowel disease in an Irish prospective cohort. *Journal of Pediatric Gastroenterology and Nutrition*. 2019;69(Supplement 1):47.

51 Daum F, Gould HB, Gold D, et al. Asymptomatic transient uveitis in children with inflammatory bowel disease. *American Journal of Diseases of Children*. 1979;133:170-171.

52 Davis P, Thomson AB, Lentle BC. Quantitative sacroiliac scintigraphy in patients with Crohn's disease. *Arthritis & Rheumatism*. Comparative Study. 1978;21:234-237.

53 Demir I, Kahveci S, Aksoy B, et al. Overlap Syndrome in Children: A Single Center Exprience. *Journal of Pediatric Gastroenterology and Nutrition*. 2023;76(Supplement 1):838.

54 Derfalvi B, Bozsaki G, Szabo D, et al. Joint involvement in pediatric crohn's disease is related to higher disease activity and worse quality of life. *Arthritis and Rheumatology*. 2014;10):S995-S996.

55 Deshayes P, Houdent C, Hecketsweiler P. [Rheumatic manifestations of Crohn's disease. A national survey]. *Revue du Rhumatisme et des Maladies Osteo-Articulaires*. 1976;43:541-551.

56 Dilillo D, Batticciotto A, Nugnes M, et al. High prevalence of entheseal involvement in paediatric inflammatory bowel disease patients: An ultrasonographic study. *Digestive and Liver Disease*. 2014;46(SUPPL 3):e78.

57 Dupre A, Collins M, Nocturne G, et al. Articular manifestations in patients with inflammatory bowel disease treated with vedolizumab. *Rheumatology*. Observational Study. 2020;59:3275-3283.

58 El-Shabrawi MH, Tarek S, Abou-Zekri M, et al. Hepatobiliary manifestations in children with inflammatory bowel disease: A single-center experience in a low/middle income country. *World Journal of Gastrointestinal Pharmacology and Therapeutics*. 2020;11:48-58.

59 Ernst BB, Lowder CY, Meisler DM, et al. Posterior segment manifestations of inflammatory bowel disease. *Ophthalmology*. 1991;98(8):1272-1280.

60 Farahmand F, Ahmadi M, Khodadad A, et al. IgG4 subclass and gamma-glutamyl transferase in children with ulcerative colitis with primary sclerosing cholangitis and without sclerosing cholangitis. *Clinical & Experimental Hepatology*. 2019;5:285-288.

61 Farinelli E, Pagnin AF, Silva RPL, et al. Epidemiological profile of Crohn's disease and ulcerative colitis in a Brazilian single centre. *Journal of Crohn's and Colitis*. 2017;11(Supplement 1):S499.

62 Fedor I, Zold E, Barta Z. Temporal relationship of extraintestinal manifestations in inflammatory bowel disease. *Journal of Clinical Medicine*. 2021;10(24) (no pagination).

63 Felekis T, Katsanos K, Kitsanou M, et al. Spectrum and frequency of ophthalmologic manifestations in patients with inflammatory bowel disease: a prospective single-center study. *Inflammatory Bowel Diseases*. Clinical Trial

Comparative Study. 2009;15:29-34.

64 Feler AY, Cohen S. High body mass index and anemia at diagnosis are predictors of extra-intestinal manifestations in children with inflammatory bowel disease. *Journal of Pediatric Gastroenterology and Nutrition*. 2018;66(Supplement 2):612-613.

65 Fraga M, Fournier N, Safroneeva E, et al. Primary sclerosing cholangitis in the Swiss Inflammatory Bowel Disease Cohort Study: Prevalence, risk factors, and long-term follow-up. *European Journal of Gastroenterology and Hepatology*. 2017;29(1):91-97.

66 Fumery M, Pariente B, Sarter H, et al. Long-term outcome of pediatric-onset Crohn's disease: A population-based cohort study. *Digestive and Liver Disease*. 2019;51(4):496-502.

67 Gerenli N, Sozeri B. Enthesithis, obscured symptom in paediatric IBD? *Turkish Journal of Gastroenterology*. 2019;30(Supplement 3):S433.

68 Ghersin I, Katz LH, Daher S, et al. Comorbidities in adolescents with inflammatory bowel disease: Findings from a population-based cohort. *Journal of Crohn's and Colitis*. 2018;12(Supplement 1):S493.

69 Ghione S, Riva S, Cirillo F, et al. Inflammatory bowel disease associated with inflammatory cholangitis had a distinct phenotype in children. *Digestive and Liver Disease*. 2012;44(SUPPL 4):S259.

70 Giani T, Di Maurizio M, Lionetti P, et al. Prevalence of subclinical sacroiliitis in young patients with inflammatory bowel disease revealed by entero-MRI. *Arthritis and Rheumatology*. 2019;71(Supplement 10):2033-2034.

71 Giani T, Filistrucchi V, Basile M, et al. Prevalence of subclinical sacroiliitis in young patients with inflammatory bowel disease revealed by entero-MRI. *Annals of the Rheumatic Diseases*. 2019;78(Supplement 2):974-975.

72 Gilat T, Lilos P, Benaroya Y, et al. Risk factors in ulcerative colitis. *Digestion*. 1976;14:400-408.

73 Gomollon F, Seoane-Mato D, Montoro MA, et al. Validation of screening criteria for spondyloarthritis in patients with inflammatory bowel disease in routine clinical practice. *Digestive & Liver Disease*. Multicenter Study

Observational Study. 2022;54:755-762.

74 Goyal A, Hyams JS, Lerer T, et al. Prospective multicenter pediatric cohort study: Patients with liver enzyme elevations in the first 3 months after diagnosis of inflammatory bowel disease are at increased risk of chronic liver disease. *Gastroenterology*. 2012;142(5 SUPPL 1):S37.

75 Goyal A, Bass J. Safety and Efficacy of Combining Biologicals in Children with Inflammatory Bowel Disease. *Inflammatory Bowel Diseases*. 2016;26(Supplement 1):S75.

76 Greuter T, Biedermann L, Scharl M, et al. Frequency, chronological order of appearance and anti-TNF treatment of extraintestinal manifestations in pediatric patients with inflammatory bowel disease. *United European Gastroenterology Journal*. 2016;4(5 Supplement 1):A461.

77 Grzybowska-Chlebowczyk U, Wiecek S, Wos H, et al. Liver function and bile duct disorders in the course of inflammatory bowel disease in children. *Gastroenterologia Polska*. 2008;15(2):107-110.

78 Gupta N, Bostrom AG, Kirschner BS, et al. Gender differences in presentation and course of disease in pediatric patients with Crohn disease. *Pediatrics*. Comparative Study

Research Support, N.I.H., Extramural

Research Support, Non-U.S. Gov't. 2007;120:e1418-1425.

79 Gutierrez A, Zapater P, Ricart E, et al. Immigrant IBD Patients in Spain Are Younger, Have More Extraintestinal Manifestations and Use More Biologics Than Native Patients. *Frontiers in Medicine*. 2022;9 (no pagination).

80 Haapamaki J, Roine RP, Turunen U, et al. Increased risk for coronary heart disease, asthma, and connective tissue diseases in inflammatory bowel disease. *Journal of Crohn's & colitis*. Research Support, Non-U.S. Gov't. 2011;5:41-47.

81 Hammoudeh M, Alkaabi S, Sharma M, et al. Rheumatic manifestations of inflammatory bowel diseases, study from middle east. *Annals of the Rheumatic Diseases*. 2017;76(Supplement 2):1309.

82 Hattar LN, Abraham BP, Malaty HM, et al. Inflammatory bowel disease characteristics in Hispanic children in Texas. *Inflammatory Bowel Diseases*. 2012;18(3):546-554.

83 Hiller A, Biedermann L, Fournier N, et al. The appearance of joint manifestations in the Swiss inflammatory bowel disease cohort. *PLoS ONE [Electronic Resource]*. Research Support, Non-U.S. Gov't. 2019;14:e0211554.

84 Hirche TO, Russler J, Braden B, et al. Sonographic detection of perihepatic lymphadenopathy is an indicator for primary sclerosing cholangitis in patients with inflammatory bowel disease. *International Journal of Colorectal Disease*. Clinical Trial. 2004;19:586-594.

85 Holowach J, Thurston DL. Chronic ulcerative colitis in childhood. *Journal of Pediatrics*. 1956;48(3):279-291.

86 Hopkins DJ, Horan E, Burton IL, et al. Ocular disorders in a series of 332 patients with Crohn's disease. *British Journal of Ophthalmology*. 1974;58:732-737.

87 Hsu YC, Wu TC, Lo YC, et al. Gastrointestinal complications and extraintestinal manifestations of inflammatory bowel disease in Taiwan: A population-based study. *Journal of the Chinese Medical Association: JCMA*. 2017;80:56-62.

88 Jacobs M, Winkelman EI, Farmer RG. Relationship of erythema nodosum to other manifestations of Crohn's disease. *Cleveland Clinic Quarterly*. 1977;44:145-148.

89 Jalan KN, Prescott RJ, Walker RJ, et al. Arthropathy, ankylosing spondylitis, and clubbing of fingers in ulcerative colitis. *Gut*. 1970;11:748-754.

90 Jang HJ, Kang B, Kim JE, et al. Extraintestinal Manifestation and Its Relation with the Clinical Factors of Pediatric Inflammatory Bowel Disease. *Gut and Liver*. 2017;11(Supplement 6):240.

91 Jang HJ, Kang B, Kim JE, et al. Extraintestinal manifestation and its relation with the clinical factors of paediatric inflammatory bowel disease. *Journal of Pediatric Gastroenterology and Nutrition*. 2018;66(Supplement 2):539.

92 Jangi S, Ruan A, Korzenik J, et al. South Asian Patients with Inflammatory Bowel Disease in the United States Demonstrate More Fistulizing and Perianal Crohn Phenotype. *Inflammatory Bowel Diseases*. 2020;26(12):1933-1942.

93 Jiang CB. Impact of clinical manifestations and outcome of ulcerative colitis with primary sclerosing cholangitis in children. *Pediatrics & Neonatology*. Editorial. 2021;62:461-462.

94 Jolving LR, Zegers FD, Lund K, et al. Children and Adolescents Diagnosed With Inflammatory Bowel Disease Are at Increased Risk of Developing Diseases With a Possible Autoimmune Pathogenesis. *Inflammatory Bowel Diseases*. 2024;20:20.

95 Jose FA, Heyman MB, Ferrell L, et al. Clinical course of pediatric patients with primary sclerosing cholangitis. *Gastroenterology*. 2009;136(5 SUPPL 1):A505-A506.

96 Kalubowila U, Liyanaarachchi T, Galketiya KB, et al. Epidemiology and clinical course of inflammatory bowel disease in the Central Province of Sri Lanka: A hospital-based study. *JGH Open*. 2018;2(4):129-133.

97 Kappelman M, Galanko J, Porter C, et al. Young faculty clinical investigator award association of pediatric inflammatory bowel disease with other immune-mediated diseases. *Journal of Pediatric Gastroenterology and Nutrition*. 2009;49(SUPPL 1):E67-E68.

98 Kappelman MD, Galanko JA, Porter CQ, et al. Association of paediatric inflammatory bowel disease with other immune-mediated diseases. *Archives of Disease in Childhood*. Research Support, N.I.H., Extramural. 2011;96:1042-1046.

99 Kelly P, Patchett S, McCloskey D, et al. Sclerosing cholangitis, race and sex. *Gut*. 1997;41:688-689.

100 Kumagai J, Taida T, Ogasawara S, et al. Clinical characteristics and outcomes of primary sclerosing cholangitis and ulcerative colitis in Japanese patients. *PLoS ONE [Electronic Resource]*. Research Support, Non-U.S. Gov't. 2018;13:e0209352.

101 Lakatos L, Pandur T, David G, et al. Association of extraintestinal manifestations of inflammatory bowel disease in a province of western Hungary with disease phenotype: results of a 25-year follow-up study. *World Journal of Gastroenterology*. 2003;9:2300-2307.

102 Lam S, Squires JE, Rosen MJ, et al. Circulating interleukin 10 (IL10) and mip-3alpha (CCL20) segregates liver disease from transient elevation of liver enzymes in a discovery cohort of pediatric ulcerative colitis-analysis from the protect study. *Hepatology*. 2018;68(Supplement 1):1044A.

103 Lazdr D, Tornea D, Girboni L, et al. Crohn's disease: Phenotypic characteristics in western part of Romania. *Journal of Gastrointestinal and Liver Diseases*. 2017;26(Supplement 3):72.

104 Ledder O, Hardikar W, Catto-Smith AG, et al. Inflammatory bowel disease in children under 6 years old. *Journal of Gastroenterology and Hepatology*. 2010;25(SUPPL S3):A96.

105 Lee HJ. Ocular inflammations in patients with inflammatory bowel disease in Korea: A prospective single center cross sectional study. *Journal of Clinical Immunology*. 2016;36(3):248.

106 Lepisto A, Karkkainen P, Jarvinen HJ. Prevalence of primary sclerosing cholangitis in ulcerative colitis patients undergoing proctocolectomy and ileal pouch-anal anastomosis. *Inflammatory Bowel Diseases*. 2008;14:775-779.

107 Lev-Tzion R, Warner N, Muise A, et al. Phenotype, genetics and outcomes in an infantile-onset IBD cohort. *Journal of Pediatric Gastroenterology and Nutrition*. 2018;66(Supplement 2):12.

108 Levitt MD, Ritchie JK, Lennard-Jones JE, et al. Pyoderma gangrenosum in inflammatory bowel disease. *British Journal of Surgery*. Research Support, Non-U.S. Gov't. 1991;78:676-678.

109 Lopez RN, Evans HM, Appleton L, et al. Prospective Incidence of Paediatric Inflammatory Bowel Disease in New Zealand in 2015: Results from the Paediatric Inflammatory Bowel Disease in New Zealand (PINZ) Study. *Journal of Pediatric Gastroenterology and Nutrition*. 2018;66(5):e122-e126.

110 Louis EJ, Lofberg R, Reinisch W, et al. Sustained resolution of extraintestinal manifestations in patients with crohn's disease receiving adalimumab. *United European Gastroenterology Journal*. 2016;4(5 Supplement 1):A442-A443.

111 Louis EJ, Reinisch W, Schwartz DA, et al. Adalimumab Reduces Extraintestinal Manifestations in Patients with Crohn's Disease: A Pooled Analysis of 11 Clinical Studies. *Advances in Therapy*. Research Support, Non-U.S. Gov't. 2018;35:563-576.

112 Ludvigsson JF, Busch K, Olen O, et al. Prevalence of paediatric inflammatory bowel disease in Sweden: a nationwide population-based register study. *BMC Gastroenterology*. 2017;17:23.

113 Lustosa A, Ribeiro H, Nobrega J, et al. Primary Sclerosing Cholangitis and Ulcerative Colitis: A Correlation to Be Considered in Childhood. *Inflammatory Bowel Diseases*. 2017;23(Supplement 1):S67.

114 Malaty H, Abraham B, Mehta S, et al. Effect of gender on the natural history of ulcerative colitis in pediatric population: A 20 year population-based study. *American Journal of Gastroenterology*. 2011;106(SUPPL 2):S465.

115 Malaty HM, Abraham BP, Mehta S, et al. The natural history of ulcerative colitis in pediatric population: A 20 year population-based study. *Gastroenterology*. 2011;140(5 SUPPL 1):S512-S513.

116 Malaty HM, Mehta S, Garnett EA, et al. Arthritis associated with inflammatory bowel disease in childhood: A 15 years study. *Gastroenterology*. 2011;140(5 SUPPL 1):S736-S737.

117 Malaty HM, Abraham B, Mehta S, et al. Prevalence and risk factors of extra intestinal manifestations in the pediatric inflammatory bowel disease cohort. *Gastroenterology*. 2012;142(5 SUPPL 1):S666.

118 Malaty HM, Garnett EA, Ferry GD. Effect of biologics on the long term outcomes of inflammatory bowel disease in children: A 25-year follow-up study. *Gastroenterology*. 2013;144(5 SUPPL 1):S708.

119 Manguso F, Sanges M, Staiano T, et al. Cigarette smoking and appendectomy are risk factors for extraintestinal manifestations in ulcerative colitis. *American Journal of Gastroenterology*. Research Support, Non-U.S. Gov't. 2004;99:327-334.

120 Marin-Jimenez I, Gisbert JP, Perez-Calle JL, et al. [Two-year incidence of new immune-mediated inflammatory diseases in patients with inflammatory bowel disease: A study in the AQUILES cohort]. *Gastroenterologia y Hepatologia*. Multicenter Study

Observational Study

Research Support, Non-U.S. Gov't. 2015;38:569-574.

121 Martinez J, Muncunill GP, Cuerva BG, et al. Clinical patterns and outcomes of Very-Early-Onset Inflammatory Bowel Disease (VEOIBD)-A single centre experience. *Journal of Pediatric Gastroenterology and Nutrition*. 2018;66(Supplement 2):562.

122 Martinez-Osorio J, Garcia B, Gonzalez J, et al. Very-early-onset inflammatory bowel disease (VEOIBD); Clinical presentation, response to therapy and prognosis. *Journal of Pediatric Gastroenterology and Nutrition*. 2017;65(Supplement 1):S12.

123 McErlane F, Gillon C, Irvine T, et al. Arthropathy in paediatric inflammatory bowel disease: a cross-sectional observational study. *Rheumatology*. Letter. 2008;47:1251-1252.

124 Mendes FD, Levy C, Enders FB, et al. Abnormal hepatic biochemistries in patients with inflammatory bowel disease. *American Journal of Gastroenterology*. Research Support, Non-U.S. Gov't. 2007;102:344-350.

125 Mendoza JL, Lana R, Taxonera C, et al. [Extraintestinal manifestations in inflammatory bowel disease: differences between Crohn's disease and ulcerative colitis]. *Medicina Clinica*. 2005;125:297-300.

126 Mendoza JL, Lana R, Martin MC, et al. FcRL3 gene promoter variant is associated with peripheral arthritis in Crohn's disease. *Inflammatory Bowel Diseases*. Clinical Trial

Research Support, Non-U.S. Gov't. 2009;15:1351-1357.

127 Merrick V, Henderson P, Drummond H, et al. The prevalence of autoimmune diseases in a nationwide paediatric inflammatory bowel disease cohort. *Journal of Crohn's and Colitis*. 2015;9(SUPPL 1):S30-S31.

128 Merrick VM, Henderson P, Drummond H, et al. Juvenile idiopathic arthritis and other autoimmune diseases in a nationwide paediatric inflammatory bowel disease cohort. *Archives of Disease in Childhood*. 2015;100(SUPPL 3):A146.

129 Mocelin V, Nisihara RM, Utiyama SR, et al. Anti-CCP Antibodies and Rheumatological Findings in Brazilian Patients with Crohn's Disease. *Digestion*. 2015;91:303-306.

130 Moller C, Linden G. Ulcerative colitis in Finland. I. Cases treated at central hospitals, 1956-1967. *Diseases of the Colon & Rectum*. 1971;14:259-263.

131 Moravvej H, Razavi GM, Farshchian M, et al. Cutaneous manifestations in 404 Iranian patients with inflammatory bowel disease: a retrospective study. *Indian Journal of Dermatology, Venereology & Leprology*. Comparative Study. 2008;74:607-610.

132 Morl M. [Hepatic reaction in ulcerative colitis and Crohn's disease (author's transl)]. *Medizinische Klinik*. 1975;70:670-673.

133 Morlock G, Bataille R, Blotman F, et al. [Rheumatismal manifestations of coloenteropathies]. *Revue du Rhumatisme et des Maladies Osteo-Articulaires*. 1976;43:669-677.

134 Mosebach S, Tromm A, Wittenborg A, et al. [Rheumatoid disorders in Crohn disease and ulcerative colitis. Dominance of non-inflammatory factors]. *Leber, Magen, Darm*. Research Support, Non-U.S. Gov't. 1995;25:76, 79-85.

135 Nambu R, Hagiwara SI, Kubota M, et al. Difference between early onset and late-onset pediatric ulcerative colitis. *Pediatrics International*. 2016;58(9):862-866.

136 Nuij VJAA, Zelinkova Z, Rijk MCM, et al. Phenotype of inflammatory bowel disease at diagnosis in the Netherlands: A population-based inception cohort study (the Delta Cohort). *Inflammatory Bowel Diseases*. 2013;19(10):2215-2222.

137 O'Toole A, Alakkari A, Keegan D, et al. Primary sclerosing cholangitis and disease distribution in inflammatory bowel disease. *Clinical Gastroenterology & Hepatology*. 2012;10:439-441.

138 Okada M, Yao T, Fuchigami T, et al. Anatomical involvement and clinical features in 91 Japanese patients with Crohn's disease. *Journal of Clinical Gastroenterology*. 1987;9:165-171.

139 Olbjorn C, Rove JB, Jahnsen J. Combination of Biological Agents in Moderate to Severe Pediatric Inflammatory Bowel Disease: A Case Series and Review of the Literature. *Pediatric Drugs*. 2020;22(4):409-416.

140 Olsson R, Danielsson A, Jarnerot G, et al. Prevalence of primary sclerosing cholangitis in patients with ulcerative colitis. *Gastroenterology*. Research Support, Non-U.S. Gov't. 1991;100:1319-1323.

141 Palm O, Moum B, Jahnsen J, et al. The prevalence and incidence of peripheral arthritis in patients with inflammatory bowel disease, a prospective population-based study (the IBSEN study). *Rheumatology*. Research Support, Non-U.S. Gov't. 2001;40:1256-1261.

142 Palm O, Moum B, Ongre A, et al. Prevalence of ankylosing spondylitis and other spondyloarthropathies among patients with inflammatory bowel disease: a population study (the IBSEN study). *Journal of Rheumatology*. Research Support, Non-U.S. Gov't. 2002;29:511-515.

143 Papamichael K, Tsirogianni A, Papasteriades C, et al. Low prevalence of antibodies to cyclic citrullinated peptide in patients with inflammatory bowel disease regardless of the presence of arthritis. *European Journal of Gastroenterology & Hepatology*. 2010;22:705-709.

144 Pasternak B, Grom A, Yazigi N, et al. Suppurative peripheral arthritis in inflammatory bowel disease. *Journal of Pediatric Gastroenterology & Nutrition*. Case Reports. 2007;45:117-120.

145 Patil K, Alsharief A, Al-Rayahi J, et al. MRCP in primary sclerosing cholangitis in children with inflammatory bowel disease: A retrospective study. *Pediatric Radiology*. 2019;49(Supplement 2):S295.

146 Peeters H, Vander Cruyssen B, Laukens D, et al. Radiological sacroiliitis, a hallmark of spondylitis, is linked with CARD15 gene polymorphisms in patients with Crohn's disease. *Annals of the Rheumatic Diseases*. Research Support, Non-U.S. Gov't. 2004;63:1131-1134.

147 Peeters H, Vander Cruyssen B, Mielants H, et al. Clinical and genetic factors associated with sacroiliitis in Crohn's disease. *Journal of Gastroenterology & Hepatology*. Multicenter Study

Research Support, Non-U.S. Gov't. 2008;23:132-137.

148 Penn KA, Whittle DO, Lee MG. Inflammatory bowel disease in Jamaica. *Annals of Gastroenterology*. 2013;26(3):239-242.

149 Platt JW, Schleainger BE, Beneon PF. Ulcerative colites in childhood. A study of its natural history. *Quarterly journal of medicine*. 1960;29(114):257-277.

150 Podberesky D, Sullivan J, Salisbury S, et al. Prevalence of penetrating and stricturing complications, and extraintestinal manifestations detected by CT enterography in pediatric inflammatory bowel disease patients. *Pediatric Radiology*. 2010;40(6):1093.

151 Porfito C, Orlando F, Laurenza C, et al. Articular involvement in pediatric inflammatory bowel disease (IBD). *Pediatric Rheumatology Conference: 25th European Paediatric Rheumatology Congress, PReS*. 2018;16.

152 Powell FC, Schroeter AL, Su WP, et al. Pyoderma gangrenosum: a review of 86 patients. *Quarterly Journal of Medicine*. 1985;55:173-186.

153 Prathapan KM, Rivers CR, Kim SC, et al. Peripheral Blood Eosinophilia Is a Biomarker of Long-Term Severity in Pediatric-Onset Inflammatory Bowel Disease Patients. *Gastroenterology*. 2019;156(6 Supplement 1):S-392-S-393.

154 Prathapan KM, Ramos Rivers C, Anderson A, et al. Peripheral Blood Eosinophilia and Long-term Severity in Pediatric-Onset Inflammatory Bowel Disease. *Inflammatory Bowel Diseases*. Research Support, U.S. Gov't, Non-P.H.S. 2020;26:1890-1900.

155 Pusateri A, Crandall W, Dotson J, et al. Characterizing frequency, pattern and etiology of elevated liver enzymes in pediatric inflammatory bowel disease patients: A 3-year retrospective, single-center review. *Inflammatory Bowel Diseases*. 2013;19(SUPPL 1):S95.

156 Qari YA. Clinical characteristics of Crohn's disease in a cohort from Saudi Arabia. *Saudi Journal of Medicine and Medical Sciences*. 2022;10(1):56-62.

157 Ramzan NN, Leighton JA, Heigh RI, et al. Clinical signifacance of granuloma in Crohn's disease. *Inflammatory Bowel Diseases*. 2002;8(3):168-173.

158 Rasheed S, Stimec J, Ranson M, et al. Inflammatory and non-inflammatory musculoskeletal findings on Magnetic Resonance Enterography (MRE) in paediatric IBD patients. *Pediatric Radiology*. 2015;45(2 SUPPL 1):S356.

159 Rasmussen HH, Fallingborg J, Mortensen PB, et al. Primary sclerosing cholangitis in patients with ulcerative colitis. *Scandinavian Journal of Gastroenterology*. 1992;27:732-736.

160 Reilly E, Edwards C, Mackay K. Anti-tnf therapy induced arthritis in patients with inflammatory bowel disease. *Rheumatology (United Kingdom)*. 2015;54(Supplement 1):i94.

161 Ribaldone DG, Garavagno M, Pellicano R, et al. Prevalence and prognostic value of hepatic histological alterations in patients with Crohn's disease. *Scandinavian Journal of Gastroenterology*. 2015;50:1463-1468.

162 Ribaldone DG, Pellicano R, Actis GC. The gut and the inflammatory bowel diseases inside-out: Extra-intestinal manifestations. *Minerva Gastroenterologica e Dietologica*. 2019;65(4):309-318.

163 Rodriguez-Perez N, Aguinaga-Barrilero A, Gorrono-Echebarria MB, et al. Analysis of Crohn's disease-related CARD15 polymorphisms in Spanish patients with idiopathic uveitis. *Disease Markers*. Research Support, Non-U.S. Gov't. 2008;24:111-117.

164 Ronnblom A, Holmstrom T, Tanghoj H, et al. Hepatobiliary diseases in a prospective population based cohort with inflammatory bowel diseases (ICURE). *United European Gastroenterology Journal*. 2014;2(1 SUPPL 1):A204.

165 Roth N, Biedermann L, Fournier N, et al. Occurrence of skin manifestations in patients of the Swiss Inflammatory Bowel Disease Cohort Study. *PLoS ONE [Electronic Resource]*. Research Support, Non-U.S. Gov't. 2019;14:e0210436.

166 Roth R, Vavricka S, Scharl M, et al. The impact of colectomy on the course of extraintestinal manifestations in Swiss inflammatory bowel disease cohort study patients. *United European Gastroenterology Journal*. 2021;9(7):773-780.

167 Ruemmele F, Pigneur B. Is there a correlation between anti-tumor necrosis factor trough levels and the occurrence of paradoxical cutaneous and articular manifestations in children with inflammatory bowel disease (IBD)? *Journal of Pediatric Gastroenterology and Nutrition*. 2016;63(Supplement 2):S76.

168 Sahli H, Fekih M, Landolsi F, et al. [Articular manifestations in Crohn's disease. A prospective study of 68 cases]. *Tunisie Medicale*. 2006;84:532-536.

169 Saigot T, Bousquet O, Delas N, et al. [Cutaneous and mucosal manifestations of Crohn's disease]. *Annales de Medecine Interne*. Case Reports. 1977;128:527-531.

170 Sams WM, Jr., Winkelmann RK. The association of erythema nodosum with ulcerative colitis. *Southern Medical Journal*. 1968;61:676-679.

171 Schwartz D, Louis EJ, Lofberg R, et al. Sustained resolution of extraintestinal manifestations in patients with Crohn's disease receiving adalimumab. *American Journal of Gastroenterology*. 2016;111(Supplement 1):S305.

172 Sdepanian VL, Lopes LHC, Venturieri MO, et al. Infliximab and adalimumab in pediatric inflammatory bowel disease: Clinical practice at a tertiary care IBD referral center for 16 years. *Journal of Pediatric Gastroenterology and Nutrition*. 2021;72(SUPPL 1):727.

173 Shaul E, Conrad M, Dawany N, et al. Canakinumab use in autoinflammatory very early onset-inflammatory bowel disease. *Journal of Pediatric Gastroenterology and Nutrition*. 2021;73(1 SUPPL 1):S240-S241.

174 Shaul E, Patel T, Aleynick NDD, et al. A Targeted Approach with Canakinumab in Children with Veo-Ibd with Autoinflammatory Phenotype. *Gastroenterology*. 2021;160(6 Supplement):S-146.

175 Shentova R, Panteleeva E, Zhelev C, et al. Extraintestinal manifestations in Bulgarian pediatric patients with inflammatory bowel disease a single center experience. *Journal of Crohn's and Colitis*. 2014;8(SUPPL 2):S403-S404.

176 Shentova-Eneva R, Baycheva M, Hadjiiski P, et al. Extraintestinal manifestations in paediatric patients with inflammatory bowel diseases. *Journal of Pediatric Gastroenterology and Nutrition*. 2019;68(Supplement 1):600.

177 Sheybani E, Greer ML. Incidental extra-intestinal findings on magnetic resonance enterography in children with inflammatory bowel disease. *Pediatric Radiology*. 2014;44(SUPPL 1):S217.

178 Shitrit ABG, Koslowsky B, Kori M, et al. Inflammatory bowel disease: An emergent disease among ethiopian jews migrating to Israel. *Inflammatory Bowel Diseases*. 2015;21(3):631-635.

179 Shivashankar R, Loftus EV, Jr., Tremaine WJ, et al. Incidence of spondyloarthropathy in patients with Crohn's disease: a population-based study. *Journal of Rheumatology*. Research Support, N.I.H., Extramural

Research Support, Non-U.S. Gov't. 2012;39:2148-2152.

180 Shrestha S, Brand JS, Jaras J, et al. Association between inflammatory bowel disease and spondyloarthropathies: Findings from a nationwide study in Sweden. *United European Gastroenterology Journal*. 2021;9(SUPPL 8):422-423.

181 Shrestha S, Brand JS, Järås J, et al. Association Between Inflammatory Bowel Disease and Spondyloarthritis: Findings from a Nationwide Study in Sweden. *J Crohns Colitis*. 2022;16:1540-1550.

182 Sladek MK, Herman RB, Herman-Sucharska I, et al. Primary sclerosing cholangitis in pediatrics newly-onest Crohn's disease is associated with male predominance, perianal lesions and abberant serological response. *United European Gastroenterology Journal*. 2013;1(1 SUPPL 1):A3.

183 Spencer E, Bergstein S, Dolinger M, et al. Single Center Experience with Upadacitinib for Refractory Adolescent Inflammatory Bowel Disease. *Journal of Pediatric Gastroenterology and Nutrition*. 2023;77(1 Supplement 1):S70-S71.

184 Sridhar S, Maltz RM, Boyle B, et al. Dermatological Manifestations in Pediatric Patients with Inflammatory Bowel Diseases on Anti-TNF Therapy. *Inflammatory Bowel Diseases*. Evaluation Study. 2018;24:2086-2092.

185 Subramaniam K, Tymms K, Shadbolt B, et al. Spondyloarthropathy in inflammatory bowel disease patients on TNF inhibitors. *Internal Medicine Journal*. 2015;45:1154-1160.

186 Szakos E, Monus A, Kosaras E, et al. Skin symptoms in pediatric patients with inflammatory bowel diseases. *Journal of Pediatric Gastroenterology and Nutrition*. 2019;69(Supplement 1):67.

187 Takeuchi M, Tomomasa T, Yasunaga H, et al. Descriptive epidemiology of children hospitalized for inflammatory bowel disease in Japan: Inpatient database analysis. *Pediatrics International*. 2015;57(3):443-448.

188 Templier C, Sarter H, Turck D, et al. Cumulative incidence and associated factors of muco-cutaneous manifestations in paediatric-onset Crohn's disease: A population-based study. *United European Gastroenterology Journal*. 2014;2(1 SUPPL 1):A227.

189 Templier C, Sarter H, Turck D, et al. Incidence and associated factors of cutaneous manifestations in paediatric-onset crohn's disease: A population-based study. *Gastroenterology*. 2015;148(4 SUPPL 1):S248.

190 Templier C, Sarter H, Turck D, et al. Incidence and risk factors of cutaneous manifestations in pediatric-onset Crohn'sdisease: A population-based study. *Journal of Crohn's and Colitis*. 2015;9(SUPPL 1):S25.

191 Thakkar K, Patel A, Thibodeaux C, et al. Characteristics of pediatric IBD in Texas. *Journal of Pediatric Gastroenterology and Nutrition*. 2010;51(SUPPL 2):E87.

192 Toledo-Maurino JJ, Yamamoto-Furusho JK. Drug allergy is associated with the development of extraintestinal manifestations in patients with ulcerative colitis. *European Annals of Allergy & Clinical Immunology*. 2020;52:35-38.

193 Topaloğlu Demir F, Kocatürk E, Yorulmaz E, et al. Mucocutaneous manifestations of inflammatory bowel disease in Turkey. *J Cutan Med Surg*. 2014;18:397-404.

194 Toy E, Balasubramanian S, Selmi C, et al. The prevalence, incidence and natural history of primary sclerosing cholangitis in an ethnically diverse population. *BMC Gastroenterology*. Research Support, N.I.H., Extramural. 2011;11:83.

195 Tse CS, Deepak P, De La Fuente J, et al. Phenotype and Clinical Course of Inflammatory Bowel Disease With Co-existent Celiac Disease. *Journal of Crohn's & colitis*. 2018;12:973-980.

196 Turkcapar N, Toruner M, Soykan I, et al. The prevalence of extraintestinal manifestations and HLA association in patients with inflammatory bowel disease. *Rheumatology International*. 2006;26:663-668.

197 Uchino M, Ikeuchi H, Matsuoka H, et al. Clinical features and management of parastomal pyoderma gangrenosum in inflammatory bowel disease. *Digestion*. 2012;85:295-301.

198 Usoltseva O, Movsisyan G, Surkov A. Primary sclerosing cholangitis in children with inflammatory bowel disease. *Journal of Pediatric Gastroenterology and Nutrition*. 2021;72(SUPPL 1):1001.

199 Vadstrup K, Alulis S, Borsi A, et al. Extraintestinal Manifestations and Other Comorbidities in Ulcerative Colitis and Crohn Disease: A Danish Nationwide Registry Study 2003-2016. *Crohn's and Colitis 360*. 2020;2(3):1-10.

200 Valentino PL, Feldman BM, Walters TD, et al. The prevalence and spectrum of abnormal liver biochemistry in a large cohort of children with inflammatory bowel disease. *Hepatology*. 2013;58(4 SUPPL 1):815A-816A.

201 Varas P, Antunez-Lay A, Bernucci JM, et al. [Erythema nodosum: Analysis of 91 hospitalized patients]. *Revista Medica de Chile*. 2016;144:162-168.

202 Vavricka SR, Rogler G, Gantenbein C, et al. Chronological Order of Appearance of Extraintestinal Manifestations Relative to the Time of IBD Diagnosis in the Swiss Inflammatory Bowel Disease Cohort. *Inflammatory Bowel Diseases*. Research Support, Non-U.S. Gov't. 2015;21:1794-1800.

203 Vavricka SR, Rechner R, Fournier N, et al. Prevalence of extraintestinal manifestations in pediatric patients with inflammatory bowel disease: Results from the Swiss IBD cohort study. *Gastroenterology*. 2015;148(4 SUPPL 1):S458-S459.

204 Vavricka S, Rechner R, Fournier N, et al. Prevalence of extraintestinal manifestations in paediatric patients with Inflammatory Bowel Disease: Results from the Swiss IBD Cohort Study. *Journal of Crohn's and Colitis*. 2015;9(SUPPL 1):S197.

205 Vavricka SR, Gubler M, Gantenbein C, et al. Anti-TNF Treatment for Extraintestinal Manifestations of Inflammatory Bowel Disease in the Swiss IBD Cohort Study. *Inflammatory Bowel Diseases*. Research Support, Non-U.S. Gov't. 2017;23:1174-1181.

206 Veloso FT, Carvalho J, Magro F. Immune-related systemic manifestations of inflammatory bowel disease: A prospective study of 792 patients. *Journal of Clinical Gastroenterology*. 1996;23(1):29-34.

207 Veres G, Derfalvi B, Bozsaki G, et al. Arthritis, sacroileal MRI and HLA-B27 in relation to PCDAI and quality of life in pediatric patients with Crohn's disease. *Journal of Crohn's and Colitis*. 2013;7(SUPPL.1):S128.

208 Vind I, Riis L, Jess T, et al. Increasing incidences of inflammatory bowel disease and decreasing surgery rates in Copenhagen City and County, 2003-2005: a population-based study from the Danish Crohn colitis database. *American Journal of Gastroenterology*. Research Support, Non-U.S. Gov't. 2006;101:1274-1282.

209 Wang MH, Mousa OY, Friton JJ, et al. Unique Phenotypic Characteristics and Clinical Course in Patients With Ulcerative Colitis and Primary Sclerosing Cholangitis: A Multicenter US Experience. *Inflammatory Bowel Diseases*. Multicenter Study

Observational Study

Research Support, N.I.H., Extramural

Research Support, Non-U.S. Gov't. 2020;26:774-779.

210 Wang Y, Ouyang Q. Ulcerative colitis in China: Retrospective analysis of 3100 hospitalized patients. *Journal of Gastroenterology and Hepatology (Australia)*. 2007;22(9):1450-1455.

211 Wei SC, Shieh MJ, Chang MC, et al. Long-term follow-up of ulcerative colitis in Taiwan. *Journal of the Chinese Medical Association: JCMA*. 2012;75:151-155.

212 Weng X, Liu L, Barcellos LF, et al. Clustering of inflammatory bowel disease with immune mediated diseases among members of a northern california-managed care organization. *American Journal of Gastroenterology*. Research Support, Non-U.S. Gov't. 2007;102:1429-1435.

213 Weng MT, Shih IL, Tung CC, et al. Association of young age and male sex with primary sclerosing cholangitis in Taiwanese patients with inflammatory bowel disease. *Intestinal Research*. 2022;20:224-230.

214 Wewer V, Gluud C, Schlichting P, et al. Prevalence of hepatobiliary dysfunction in a regional group of patients with chronic inflammatory bowel disease. *Scandinavian Journal of Gastroenterology*. Case Reports. 1991;26:97-102.

215 Wiecek S, Wojtyniak A, Pindur B, et al. Analysis of the Clinical Course of Primary Sclerosing Cholangitis in Paediatric Population-Single Center Study. *Medicina*. 2021;57:27.

216 Yamamoto-Furusho JK, Sanchez-Osorio M, Uribe M. Prevalence and factors associated with the presence of abnormal function liver tests in patients with ulcerative colitis. *Annals of Hepatology*. 2010;9:397-401.

217 Yamamoto-Furusho J, Sarmiento A, Toledo-Maurino J, et al. Clinical and sociodemographical characteristics of inflammatory bowel disease in Mexico: Multicentric nation-wide study (EPIMEX-IBD). *Journal of Crohn's and Colitis*. 2018;12(Supplement 1):S540.

218 Yamamoto-Furusho JK, Sanchez-Morales GE. Factors Associated with the Presence of Extraintestinal Manifestations in Patients with Ulcerative Colitis in a Latin American Country. *Inflammatory Intestinal Diseases*. 2020;5(4):200-204.

219 Ye BD, Yang SK, Boo SJ, et al. Clinical characteristics of ulcerative colitis associated with primary sclerosing cholangitis in Korea. *Inflammatory Bowel Diseases*. Comparative Study. 2011;17:1901-1906.

220 Yeh PJ, Chen CC, Chao HC, et al. High initial bowel resection rate and very-early-onset inflammatory bowel disease - A challenge in a low-prevalence area. *Journal of the Formosan Medical Association*. 2021;Part 3. 120(1):720-727.

221 Yeushalmy-Feler A, Cohen S. High body mass index and anaemia at diagnosis are predictors of extra-intestinal manifestations in children with inflammatory bowel disease. *Journal of Crohn's and Colitis*. 2018;12(Supplement 1):S263-S264.

222 Yilmaz S, Aydemir E, Maden A, et al. The prevalence of ocular involvement in patients with inflammatory bowel disease. *International Journal of Colorectal Disease*. Randomized Controlled Trial. 2007;22:1027-1030.

223 Yuksel I, Basar O, Ataseven H, et al. Mucocutaneous manifestations in inflammatory bowel disease. *Inflammatory Bowel Diseases*. 2009;15:546-550.

224 Yuksel I, Ataseven H, Basar O, et al. Peripheral arthritis in the course of inflammatory bowel diseases. *Digestive Diseases & Sciences*. 2011;56:183-187.

225 Zvidi I, Fraser GM, Niv Y, et al. The prevalence of inflammatory bowel disease in an Israeli Arab population. *Journal of Crohn's & colitis*. 2013;7:e159-163.

226 Adamiak T, Walkiewicz-Jedrzejczak D, Fish D, et al. Incidence, clinical characteristics, and natural history of pediatric IBD in Wisconsin: A population-based epidemiological study. *Inflammatory Bowel Diseases*. 2013;19(6):1218-1223.

227 Afarideh M, Bartoletta K, Tollefson MM. Dermatologic manifestations in pediatric patients with inflammatory bowel disease. *Pediatric Dermatology*. 2024;41:234-242.

228 Aguiar F, Fonseca R, Alves D, et al. The prevalence of sacroiliitis assessed by magnetic resonance imaging in a pediatric population with inflammatory bowel disease. *Pediatric Rheumatology Conference: 23rd Paediatric Rheumatology European Society Congress Genoa Italy*. 2017;15.

229 Al Saleem K, El Mouzan MI, Saadah OI, et al. Characteristics of pediatric ulcerative colitis in Saudi Arabia: A multicenter national study. *Annals of Saudi Medicine*. 2015;35(1):19-22.

230 Alexopoulou E, Xenophontos PE, Economopoulos N, et al. Investigative MRI cholangiopancreatography for primary sclerosing cholangitis-type lesions in children with IBD. *Journal of Pediatric Gastroenterology & Nutrition*. 2012;55:308-313.

231 Aloi M, D'Arcangelo G, Pofi F, et al. Presenting features and disease course of pediatric ulcerative colitis. *Journal of Crohn's and Colitis*. 2013;7(11):e509-e515.

232 Alreheili KM, Alsaleem KA, Almehaidib AI. Natural history and outcome of inflammatory bowel diseases in children in Saudi Arabia: A single-center experience. *Saudi Journal of Gastroenterology*. 2018;24:171-176.

233 Arcucci MS, Contreras MB, Gallo J, et al. Pediatric Inflammatory Bowel Disease: A Multicenter Study of Changing Trends in Argentina Over the Past 30 Years. *Pediatric Gastroenterology Hepatology & Nutrition*. 2022;25:218-227.

234 Ashton JJ, Coelho T, Ennis S, et al. Presenting phenotype of paediatric inflammatory bowel disease in Wessex, Southern England 2010-2013. *Acta Paediatrica, International Journal of Paediatrics*. 2015;104(8):831-837.

235 Ben Rabeh R, Ben Othman A, Bouyahya O, et al. Extraintestinal manifestations of pediatric inflammatory bowel disease: A Tunisian single-center experience. *Archives of Disease in Childhood*. 2019;104(Supplement 3):A290.

236 Bilgic Dagci AO, Chang JC, Brandon TG, et al. Clinical features, treatment patterns and short-term outcomes of children with inflammatory bowel disease evaluated in rheumatology clinic. *Clinical & Experimental Rheumatology*. 2022;40:1045-1051.

237 Bramuzzo M, Martelossi S, Torre G, et al. Clinical features and risk factors of autoimmune liver involvement in pediatric inflammatory bowel disease. *Journal of Pediatric Gastroenterology and Nutrition*. 2016;63(2):259-264.

238 Cakir M, Unal F, Dinler G, et al. Inflammatory bowel disease in Turkish children. *World Journal of Pediatrics*. 2015;11(4):331-337.

239 Cereser L, Zancan G, Giovannini I, et al. Asymptomatic sacroiliitis detected by magnetic resonance enterography in patients with Crohn's disease: prevalence, association with clinical data, and reliability among radiologists in a multicenter study of adult and pediatric population. *Clinical Rheumatology*. Multicenter Study. 2022;41:2499-2511.

240 Chandrakumar A, Loeppky R, Deneau M, et al. Inflammatory Bowel Disease in Children with Elevated Serum Gamma Glutamyltransferase Levels. *Journal of Pediatrics*. Research Support, N.I.H., Extramural

Research Support, Non-U.S. Gov't. 2019;215:144-151.e143.

241 Cohen S, Padlipsky J, Yerushalmy-Feler A. Risk factors associated with extraintestinal manifestations in children with inflammatory bowel disease. *European Journal of Clinical Nutrition*. 2020;74:691-697.

242 Colletti R, Griffiths A, Veereman G, et al. New Onset Autoimmune Disorders, Primarily Psoriasis, in Anti-Tnf Biologic Exposed Pediatric Patients - the Develop Experience. *Gastroenterology*. 2019;156(3 Supplement):S45.

243 Daniluk U, Kwiatek-Sredzinska K, Jakimiec P, et al. Liver Pathology in Children with Diagnosed Inflammatory Bowel Disease-A Single Center Experience. *Journal of Clinical Medicine*. 2021;10:17.

244 Dass R, Ruterbusch J, Thomas R, et al. Inflammatory Bowel Disease in the Pediatric Middle Eastern Population. *Journal of Pediatric Gastroenterology and Nutrition*. 2023;77(1 Supplement 1):S287-S289.

245 Deneau M, Jensen MK, Holmen J, et al. Primary sclerosing cholangitis, autoimmune hepatitis, and overlap in Utah children: epidemiology and natural history. *Hepatology*. Multicenter Study

Research Support, N.I.H., Extramural. 2013;58:1392-1400.

246 Derfalvi B, Boros KK, Szabo D, et al. Joint involvement, disease activity and quality of life in pediatric Crohn's disease - a cross-sectional study. *Pediatric Rheumatology Online Journal*. Observational Study. 2022;20:6.

247 Dimakou K, Pachoula I, Panayotou I, et al. Pediatric inflammatory bowel disease in Greece: 30-years experience of a single center. *Ann Gastroenterol*. 2015;28:81-86.

248 Dong F, Kern I, Weidner J, et al. Clinical course of new-onset Crohn's disease in children and adolescents in dependency of age, initial location, initial severity level and therapy over the period 2000-2014 based on the Saxon Pediatric IBD-Registry in Germany. *PLoS ONE*. 2023;18(6 June) (no pagination).

249 Dotson JL, Hyams JS, Markowitz J, et al. Extraintestinal manifestations of pediatric inflammatory bowel disease and their relation to disease type and severity. *Journal of Pediatric Gastroenterology and Nutrition*. 2010;51(2):140-145.

250 Dzongowski E, Miller M, Schmidt M, et al. Phenotype of Musculoskeletal Manifestations in a Canadian Inception Cohort of Pediatric Patients with Inflammatory Bowel Disease. *Journal of Pediatric Gastroenterology and Nutrition*. 2023;77(1 Supplement 1):S449-S450.

251 Fallahi GH, Moazzami K, Tabatabaeiyan M, et al. Clinical characteristics of Iranian pediatric patients with inflammatory bowel disease. *Acta Gastroenterologica Belgica*. 2009;72:230-234.

252 Gerenli N, Sozeri B. Enthesitis: an obscured extraintestinal manifestation in pediatric inflammatory bowel disease. *Turkish Journal of Pediatrics*. 2021;63:345-354.

253 Gerenli N, Sozeri B, Kalin S, et al. Sacroiliac joint involvement in children with inflammatory bowel diseases. *Northern Clinics of Istanbul*. 2022;9:57-63.

254 Ghersin I, Khateeb N, Katz LH, et al. Comorbidities in adolescents with inflammatory bowel disease: findings from a population-based cohort study. *Pediatric Research*. 2020;87:1256-1262.

255 Giani T, Bernardini A, Basile M, et al. Usefulness of magnetic resonance enterography in detecting signs of sacroiliitis in young patients with inflammatory bowel disease. *Pediatric Rheumatology Online Journal*. 2020;18:42.

256 Goyal A, Hyams JS, Lerer T, et al. Liver enzyme elevations within 3 months of diagnosis of inflammatory bowel disease and likelihood of liver disease. *Journal of Pediatric Gastroenterology & Nutrition*. Multicenter Study

Observational Study

Research Support, Non-U.S. Gov't. 2014;59:321-323.

257 Greuter T, Bertoldo F, Rechner R, et al. Extraintestinal Manifestations of Pediatric Inflammatory Bowel Disease: Prevalence, Presentation, and Anti-TNF Treatment. *Journal of Pediatric Gastroenterology & Nutrition*. Research Support, Non-U.S. Gov't. 2017;65:200-206.

258 Guariso G, Gasparetto M, Visonà Dalla Pozza L, et al. Inflammatory bowel disease developing in paediatric and adult age. *J Pediatr Gastroenterol Nutr*. 2010;51:698-707.

259 Hlouskova E, Bajer M, Bajerov K, et al. Autoimunne sclerosing cholangitis and inflammatory bowel disease in children: Experience from single University centrum. *Journal of Pediatric Gastroenterology and Nutrition*. 2019;68(Supplement 1):811.

260 Hofley P, Roarty J, McGinnity G, et al. Asymptomatic uveitis in children with chronic inflammatory bowel diseases. *Journal of Pediatric Gastroenterology & Nutrition*. Research Support, Non-U.S. Gov't. 1993;17:397-400.

261 Horton DB, Sherry DD, Baldassano RN, et al. Enthesitis is an Extraintestinal Manifestation of Pediatric Inflammatory Bowel Disease. *Annals of Paediatric Rheumatology*. 2012;1:10.

262 Isa HM, Mohamed AM, Al-Jowder HE, et al. Pediatric Crohn's Disease in Bahrain. *Oman Med J*. 2018;33:299-308.

263 Ivkovic L, Hojsak I, Trivic I, et al. IBD phenotype at diagnosis, and early disease-course in pediatric patients in Croatia: data from the Croatian national registry. *Pediatric Research*. 2020;88(6):950-956.

264 Jang HJ, Suh HR, Choi S, et al. Severe Disease Activity Based on the Paris Classification Is Associated with the Development of Extraintestinal Manifestations in Korean Children and Adolescents with Ulcerative Colitis. *Journal of Korean Medical Science*. 2021;36:e278.

265 Jang J, Lee SH, Jeong IS, et al. Clinical Characteristics and Long-term Outcomes of Pediatric Ulcerative Colitis: A Single-Center Experience in Korea. *Gut & Liver*. 2022;16:236-245.

266 Jashmi R, Compeyrot-Lacassagne S. The musculoskeletal manifestations of paediatric Inflammatory Bowel Disease (IBD): The gosh experience. *Pediatric Rheumatology Conference: 28th European Paediatric Rheumatology Congress, PReS*. 2022;20.

267 Jose FA, Garnett EA, Vittinghoff E, et al. Development of extraintestinal manifestations in pediatric patients with inflammatory bowel disease. *Inflammatory Bowel Diseases*. Research Support, N.I.H., Extramural

Research Support, Non-U.S. Gov't. 2009;15:63-68.

268 Kanavaki I, Kourti A, Matsota P, et al. Liver Pathology in Paediatric Patients with Inflammatory Bowel Disease - a Single Center's Expirience. *Journal of Pediatric Gastroenterology and Nutrition*. 2023;76(Supplement 1):861.

269 Kim K, Jang J. The Natural History of Ulcerative Colitis in a Pediatric Population: A Single Center Experience Between 1988 and 2013. *Inflammatory Bowel Diseases*. 2017;23(Supplement 1):S68.

270 Klemenak M, Zupan M, Riznik P, et al. Evolving Landscape of Paediatric Inflammatory Bowel Disease: Insights from a Decade-Long Study in North-East Slovenia on Incidence, Management, Diagnostic Delays, and Early Biologic Intervention. *Diagnostics*. 2024;14(2) (no pagination).

271 Kourti A, Fotis L, Papakonstantinou O, et al. Chronic Recurrent Multifocal Osteomyelitis and Pediatric Patients with Inflammatory Bowel Disease - the Expirience of a Greek Hospital. *Journal of Pediatric Gastroenterology and Nutrition*. 2023;76(Supplement 1):567-568.

272 Kwon Y, Kim ES, Choe YH, et al. Increased Demand for Therapeutic Drugs in Pediatric Ulcerative Colitis Patients With Extraintestinal Manifestations. *Frontiers in Pediatrics*. 2022;10:853019.

273 Lagercrantz R, Winberg J, Zetterstrom R. Extra-colonic manifestations in chronic ulcerative colitis. *Acta Paediatrica*. 1958;47:675-687.

274 Lee YA, Chun P, Hwang EH, et al. Clinical Features and Extraintestinal Manifestations of Crohn Disease in Children. *Pediatric Gastroenterology Hepatology & Nutrition*. 2016;19:236-242.

275 Lekovic Z, Radlovic N, Brdar R, et al. Clinical characteristics of idiopathic ulcerative colitis in children. *Srpski Arhiv Za Celokupno Lekarstvo*. 2011;139:170-173.

276 Levy R, Amarilyo G, Tal R, et al. Musculoskeletal Manifestations as Presenting Symptoms of Inflammatory Bowel Disease in Children and Adolescents. *Journal of Pediatrics*. 2019;209:233-235.

277 Lim A, Mews C, Forbes D, et al. Sclerosing cholangitis and autoimmune hepatitis in children with inflammatory bowel disease-the Western Australian experience. *Journal of Gastroenterology and Hepatology*. 2013;28(SUPPL 2):54-55.

278 Lindsley CB, Schaller JG. Arthritis associated with inflammatory bowel disease in children. *Journal of Pediatrics*. 1974;84:16-20.

279 Malmborg P, Idestrom M, Bjork J, et al. Prevalence and prognosis of patients with autoimmune liver disease in a population based childhood-onset inflammatory bowel disease cohort from northern Stockholm County. *Journal of Pediatric Gastroenterology and Nutrition*. 2016;62(SUPPL 1):508-509.

280 Maniscalco V, Scarallo L, Aloi M, et al. Prevalence and clinical features of chronic uveitis in pediatric inflammatory bowel diseases: A nationwide study. *Journal of Pediatric Gastroenterology & Nutrition*. 2024;09:09.

281 Mataly HM, Abraham BP, Mehta S, et al. The natural history of ulcerative colitis in a pediatric population: A follow-up population-based cohort study. *Clinical and Experimental Gastroenterology*. 2013;6(1):77-83.

282 Matar M, Rinawi F, Shamir R, et al. Hypergammaglobulinemia is a marker of extraintestinal manifestations in pediatric inflammatory bowel disease. *Turkish Journal of Gastroenterology*. Evaluation Study. 2017;28:131-134.

283 Medynska-Przeczek A, Stochel-Gaudyn A, Wedrychowicz A. Liver Point Shear Wave Elastography Relevance in Children with Crohn's Disease -Preliminary Study. *Journal of Pediatric Gastroenterology and Nutrition*. 2023;76(Supplement 1):594.

284 Merrick V, Henderson P, Drummond H, et al. Prevalence of autoimmune diseases in a nationwide paediatric inflammatory bowel disease cohort. *Journal of Pediatric Gastroenterology and Nutrition*. 2017;64(Supplement 1):516.

285 Naviglio S, Parentin F, Nider S, et al. Ocular Involvement in Children with Inflammatory Bowel Disease. *Inflammatory Bowel Diseases*. 2017;23:986-990.

286 Nemeth A, Ejderhamn J, Glaumann H, et al. Liver damage in juvenile inflammatory bowel disease. *Liver*. 1990;10:239-248.

287 Niewiem M, Buczynska A, Flak-Wancerz A, et al. Extraintestinal manifestations in paediatric-onset inflammatory bowel disease depending on disease location and activity. *Pediatria Polska*. 2019;94(3):162-169.

288 Nir O, Rinawi F, Amarilyo G, et al. Phenotypic Features and Longterm Outcomes of Pediatric Inflammatory Bowel Disease Patients with Arthritis and Arthralgia. *Journal of Rheumatology*. 2017;44:1636-1643.

289 Noble-Jamieson G, Heuschkel RB, Torrente F, et al. Colitis-associated sclerosing cholangitis in children: a single centre experience. *Journal of Crohn's & colitis*. 2013;7:e414-418.

290 Ong JC, O'Loughlin EV, Kamath KR, et al. Sclerosing cholangitis in children with inflammatory bowel disease. *Australian & New Zealand Journal of Medicine*. Case Reports

Research Support, Non-U.S. Gov't. 1994;24:149-153.

291 Ong CT, Cher Y, Kader A, et al. Paediatric inflammatory bowel disease in Singapore-an increasing trend in a multi-racial society. *Journal of Gastroenterology and Hepatology (Australia)*. 2014;29(SUPPL 3):132.

292 Ouldali N, Hugot JP, Viala J, et al. Early Arthritis Is Associated With Failure of Immunosuppressive Drugs and Severe Pediatric Crohn's Disease Evolution. *Inflammatory Bowel Diseases*. Research Support, Non-U.S. Gov't. 2018;24:2423-2430.

293 Passo MH, Fitzgerald JF, Brandt KD. Arthritis associated with inflammatory bowel disease in children. Relationship of joint disease to activity and severity of bowel lesion. *Digestive Diseases & Sciences*. Research Support, U.S. Gov't, P.H.S. 1986;31:492-497.

294 Pytrus T, Iwanczak B, Krzesiek E, et al. Mucocutaneous manifestations and complications in children with inflammatory bowel diseases. *Pediatria Wspolczesna*. 2011;13(2):86-91.

295 Rahmani P, Rasti G, Gorgi M, et al. Extraintestinal manifestation of inflammatory bowel disease and associated factors in pediatric patients. *Annals of Medicine & Surgery*. 2022;75:103363.

296 Rajwal SR, Puntis JW, McClean P, et al. Endoscopic rectal sparing in children with untreated ulcerative colitis. *Journal of Pediatric Gastroenterology & Nutrition*. 2004;38:66-69.

297 Rohani P, Zojaji R, Zeinali V, et al. Elevated liver enzyme in pediatric inflammatory bowel disease. *Journal of Pediatric Gastroenterology and Nutrition*. 2021;72(SUPPL 1):723.

298 Ronnblom A, Holmstrom T, Tanghoj H, et al. Appearance of hepatobiliary diseases in a population-based cohort with inflammatory bowel diseases (Inflammatory Bowel Disease Cohort of the Uppsala Region). *Journal of Gastroenterology & Hepatology*. Research Support, Non-U.S. Gov't. 2015;30:1288-1292.

299 Ruiz JA, Orsi M, Aliboni V, et al. Pediatric inflammatory bowel disease in a Latin-American population from Buenos Aires. Multicenter study. *Gastroenterology*. 2009;136(5 SUPPL 1):A356.

300 Rychwalski PJ, Cruz OA, Alanis-Lambreton G, et al. Asymptomatic uveitis in young people with inflammatory bowel disease. *Journal of Aapos: American Association for Pediatric Ophthalmology & Strabismus*. Comparative Study. 1997;1:111-114.

301 Sassine S, Savoie Robichaud M, Lin YF, et al. Changes in the clinical phenotype and behavior of pediatric luminal Crohn's disease at diagnosis in the last decade. *Digestive and Liver Disease*. 2022;54(3):343-351.

302 Schaefer M, Nelson A, Sanchez R. Uveitis in a Multicenter Pediatric Inflammatory Bowel Disease Population: Results from the Improvecarenow Network. *Gastroenterology*. 2018;154(6 Supplement 1):S-669.

303 Schoepfer AM, Tran VDC, Rossel JB, et al. Impact of Diagnostic Delay on Disease Course in Pediatric- versus Adult-Onset Patients with Ulcerative Colitis: Data from the Swiss IBD Cohort. *Inflammatory Intestinal Diseases*. 2022;7:87-96.

304 Seo JK, Yeon KM, Chi JG. Inflammatory bowel disease in children--clinical, endoscopic, radiologic and histopathologic investigation. *Journal of Korean Medical Science*. Comparative Study

Research Support, Non-U.S. Gov't. 1992;7:221-235.

305 Shentova-Eneva R, Baycheva M, Hadjiiski P, et al. Extraintestinal manifestations in paediatric patients with inflammatory bowel disease. *Journal of Crohn's and Colitis*. 2019;13(Supplement 1):S236-S237.

306 Sladek M, Herman RB, Herman-Sucharska I, et al. Aberrant serological response, male predominance, and higher rate of perianal lesions in pediatric newly-onset Crohn's disease with primary sclerosing cholangitis. *Journal of Crohn's and Colitis*. 2014;8(SUPPL 2):S437.

307 Sonavane AD, Sonawane P, Amarapurkar DN. Inflammatory Bowel Disease Across the Age Continuum: Similarity and Disparity. *Indian Journal of Pediatrics*. 2018;85(11):989-994.

308 Stenhammar L, Hogberg L, Lewander P. Primary sclerosing cholangitis in childhood inflammatory bowel disease. *Archives of Disease in Childhood*. Letter. 1994;71:281-282.

309 Taskin DG, Gulseren A. The evaluation of extraintestinal manifestations in children diagnosed with inflammatory bowel disease: A single-center experience. *Zeynep Kamil Medical Journal*. 2023;54(3):113-118.

310 Valentino PL, Feldman BM, Walters TD, et al. Abnormal Liver Biochemistry Is Common in Pediatric Inflammatory Bowel Disease: Prevalence and Associations. *Inflammatory Bowel Diseases*. Research Support, Non-U.S. Gov't. 2015;21:2848-2856.

311 Van Der Feen C, Ashton J, Batra A, et al. Study of abnormal liver function tests at diagnosis of paediatric inflammatory bowel disease (PIBD) in a large cohort in southern England. *Journal of Crohn's and Colitis*. 2014;8(SUPPL 2):S406.

312 Yousif M, Ritchey A, Mirea L, et al. Erythema Nodosum and Pyoderma Gangrenosum in Pediatric Inflammatory Bowel Disease. *Gastroenterology*. 2023;164(4 Supplement):S77-S78.

313 Yousif M, Ritchey A, Mirea L, et al. Erythema Nodosum and Pyoderma Gangrenosum in Pediatric Inflammatory Bowel Disease. *Gastroenterology*. 2023;164(6 Supplement):S-494.

314 Yu K, Davidson S, Binenbaum G. Uveitis in pediatric inflammatory bowel disease. *Journal of AAPOS*. 2023;27(3):153-155.

315 Zhou Y, Huang Y. Inflammatory bowel disease in Chinese children: A retrospective analysis of 49 cases. *Experimental & Therapeutic Medicine*. 2016;12:3363-3368.

316 Zong W, Patel A, Albenberg L. Phenotype of inflammatory bowel disease in Asian children. *Journal of Pediatric Gastroenterology and Nutrition Conference: North American Society for Pediatric Gastroenterology, Hepatology and Nutrition Annual Meeting, NASPGHAN*. 2019;69.
